# PET imaging of PARP expression using ^68^Ga-labelled inhibitors

**DOI:** 10.1007/s00259-023-06249-6

**Published:** 2023-05-05

**Authors:** Xiangwei Wang, Wei Liu, Ke Li, Kaiwen Chen, Simin He, Jianping Zhang, Bingxin Gu, Xiaoping Xu, Shaoli Song

**Affiliations:** 1 Department of Nuclear Medicine, Fudan University Shanghai Cancer Center, Shanghai, 200032 China; 2 Shanghai Engineering Research Center of Molecular Imaging Probes, Shanghai, 200032 China; 3 College of Chemistry and Materials Science, Shanghai Normal University, Shanghai, China

**Keywords:** ^68^Ga, PET/CT imaging, PARP, Olaparib, Tumors

## Abstract

**Purpose:**

Imaging the PARP expression using ^18^F probes has been approved in clinical trials. Nevertheless, hepatobiliary clearance of both ^18^F probes hindered their application in monitoring abdominal lesions. Our novel ^68^Ga-labelled probes aim for fewer abdominal signals while ensuring PARP targeting by optimizing the pharmacokinetic properties of radioactive probes.

**Methods:**

Three radioactive probes targeted PARP were designed, synthesized, and evaluated based on the PARP inhibitor Olaparib. These ^68^Ga-labelled radiotracers were assessed in vitro and in vivo.

**Results:**

Precursors that did not lose binding affinity for PARP were designed, synthesized, and then labelled with ^68^Ga in high radiochemical purity (> 97%). The ^68^Ga-labelled radiotracers were stable. Due to the increased expression of PARP-1 in SK-OV-3 cells, the uptake of the three radiotracers by SK-OV-3 cells was significantly greater than that by A549 cells. PET/CT imaging of the SK-OV-3 models indicated that the tumor uptake of ^68^Ga-DOTA-Olaparib (0.5 h: 2.83 ± 0.55%ID/g; 1 h: 2.37 ± 0.64%ID/g) was significantly higher than that of the other ^68^Ga-labelled radiotracers. There was a significant difference in the T/M (tumor-to-muscle) ratios between the unblocked and blocked groups as calculated from the PET/CT images (4.07 ± 1.01 vs. 1.79 ± 0.45, *P* = 0.0238 < 0.05). Tumor autoradiography revealed high accumulation in tumor tissues, further confirming the above data. PARP-1 expression in the tumor was confirmed by immunochemistry.

**Conclusion:**

As the first ^68^Ga-labelled PARP inhibitor, ^68^Ga-DOTA-Olaparib displayed high stability and quick PARP imaging in a tumor model. This compound is thus a promising imaging agent that can be used in a personalized PARP inhibitor treatment regimen.

**Supplementary Information:**

The online version contains supplementary material available at 10.1007/s00259-023-06249-6.

## Introduction

Tumors exhibit increased genomic instability as a result of endogenous genotoxic stress and exogenous genotoxic insults [1, 2]. PARP-1, one of the PARP (poly ADP-ribose polymerase) family of enzymes [3, 4], is overexpressed in tumor cells compared with normal tissue [5]. It is a critical DNA repair factor involved in a unique function in monitoring DNA damage and maintaining the integrity of the genome [6–8]. PARP-1 inhibition can sensitize cancerous cells to death by interfering with DNA repair and replication. This phenomenon of apoptosis induced by applying PARP-1 inhibitors in cells with *BRCA1/2* mutations or defects is known as synthetic lethality [9–12]. The FDA has approved many PARP inhibitors for treating patients with *BRCA*-mutated breast and advanced ovarian cancers [13, 14]. While the efficacy of PARP inhibitors for breast and ovarian cancer is promising, not all tumors benefit from this type of therapy [15]. Thus, developing procedures capable of assessing PARP expression and activity may be necessary to help determine which patients may benefit from PARP inhibitor treatment.

Although biopsy is still the current clinical gold standard, several studies have pointed out its shortcomings. Many tumors are known to be highly heterogeneous because of their increased genomic instability, and this heterogeneity is overlooked when a sample is taken from a single biopsy site [16]. In addition, obtaining reliable and high-quality biopsy tissue at many disease sites, such as the lung, brain, or pancreas, is a significantly invasive and complicated procedure. These challenges motivate to development of noninvasive imaging procedures to measure the entire burden of disease and better guide the selection of targeted therapies, including PARP inhibitors.

Due to the widespread use of PARP inhibitors to treat ovarian and breast cancer, PARP imaging may allow better therapeutic management in patients with both cancers. Recently, several groups have demonstrated the potential of PARP imaging with ^18^F-labelled PARP inhibitors in preclinical studies [15–26]. Most ^18^F-labelled PARP inhibitors are structurally related to Olaparib [16–22]. These compounds have been extensively explored and have displayed promising results against breast and pancreatic cancers [27, 28]. Since the current ^18^F-labelled PARP inhibitors have hydrophobic properties, they are mainly excreted by the liver and bile, resulting in a high accumulation of radioactivity in the abdomen. This physiological distribution may affect lesion detection significantly since the abdomen is the common metastatic site of pancreatic [29] and ovarian cancer [30, 31]. Thus, the application of ^18^F-labelled PARP inhibitors to evaluate PARP in both cancer types would be subject to certain restrictions.

^68^Ga is one of the most prominent radiometals used for PET imaging due to its excellent nuclide properties, ease of preparation, and fast and straightforward chemical labelling properties. High-quality images can be obtained within approximately 1 h after intravenous injection. In addition, the short half-life (67.71 min) of ^68^Ga effectively minimizes the irradiation dose administered to the patient and allows repetitive examinations within the same day [32].

In this work, we designed, synthesized, and profiled a series of ^68^Ga-labelled radiotracers based on Olaparib by analysing the SAR (structure–activity relationship) of Olaparib binding to PARP-1. After scale-up synthesis and characterization, the precursors were radiolabelled with ^68^Ga and evaluated in vitro and in vivo. In a PARP-1-positive ovarian cancer model, ^68^Ga-DOTA-Olaparib displayed a high potential to detect PARP-1 expression quantitatively. To our knowledge, this work represents the first radiosynthesis of ^68^Ga-PARP inhibitors and their translation for PET imaging.

## Materials and methods

A detailed compilation of all chemical and biological materials is described in the supplemental materials.

### Molecular docking studies

First, the protein crystal structure of soluble human PARP-1 at a resolution of 2.60 Å (RCSB PDB ID: 5DS3) was downloaded. Olaparib was re-docked into the binding site, and its binding pose from AutoDock Vina (version 1.1.2, The Scripps Research Institute, USA) exhibited excellent overlap with the original pose. Next, the three precursors were docked into the aforementioned 5DS3 structure. Then, the 3D binding modes of the three precursors and PARP-1 were predicted by PyMOL (version 2.4.0, Schrödinger, USA).

### Synthesis and radiolabelling

A detailed compilation of the precursors and intermediates and their syntheses is described in the supplemental materials. One of the three precursors (50 μg) was dissolved in 0.375 mL NaAc solution (1.5 M). 4 mL of ^68^GaCl_3_ elution was added to the above precursor solution. Radiolabelling was conducted at 100 °C for 10 min. The purification process is as follows:

At first, the C-18 column (SEP-PAK) was activated by sequentially injecting 10 mL of ethanol and 10 mL of ultrapure water into the column. After activation, the C-18 column was drained of solvent. Then 12 mL of sterile water was added to the labelled reaction system and diluted through the C-18 column, and the product was adsorbed onto the C-18 column. Then the final product was eluted from the C-18 column by injecting 1.5 mL of aqueous ethanol (V_ethanol_: V_water_ = 70%:30%). The radiochemical purity was evaluated by radio-TLC (radio-thin-layer chromatography) (NH_4_Ac/MeOH = 1:1) and radio-HPLC (radio-high-performance liquid chromatography).

### Surface plasmon resonance (SPR) binding assays

To investigate the affinity of three ^68^Ga-labelled radiotracers with PAPR-1 protein, we selected their corresponding precursors (DOTA-Olaparib, DOTA-GABA-Olaparib, and DOTA-(Gly)_3_-Olaparib) for the surface plasmon resonance (SPR) binding experiments with PARP-1, respectively. Furthermore, the compound Olaparib was used as a reference for affinity. The surface plasmon resonance binding assays were conducted according to the previous protocol [33]. The PARP-1 protein (human, 11,040-H08B, Recombinant (His Tag)) was from Sino Biological.

### Determination of the partition coefficient

***Log P:*** Generally, approximately 74–185 kBq of the ^68^Ga-labelled radiotracer was combined with a solvent mixture (2 mL, ultrapure water/octanol = 1/1). After 5 min of agitation in a vortex mixer, the aqueous and nonaqueous phases were separated by centrifugation at 15000 g. The radioactivity of each sampling layer (100 μL) was measured using a γ-counter. The trial was conducted five times.

***Log D***_***7.4***_***:*** Similarly, approximately 74–185 kBq of the ^68^Ga-labelled radiotracer was combined with a solvent mixture (2 mL, PBS (pH = 7.4)/octanol = 1/1). The partition coefficient experiment was repeated, as mentioned previously.

### Radiochemical stability

In saline, mouse plasma, and human plasma, the in vitro stability of the ^68^Ga-labelled radiotracers was evaluated. 0.2 mL of one of the three ^68^Ga-labelled radiotracers (0.74–2.96 MBq) was incubated at room temperature with 0.8 mL of saline for 0, 1, 2, and 3 h. Each combination was then evaluated by radio-HPLC to assess the stability of the ^68^Ga-labelled radiotracers.

Additionally, in an EP tube coated with an anticoagulant, 0.2 mL of one of the three ^68^Ga-labelled radiotracers (37–74 MBq) was added to 0.5–1.0 mL of fresh mouse cardiac plasma. The mixture remained at room temperature. At 0, 1, 2, and 3 h after incubation, 0.2 mL of the above solution was added to acetonitrile (0.3 mL) to precipitate plasma proteins. Then, 300 µL of water was added, and the mixture was swirled. The plasma proteins were then separated by centrifugation at 12000 g for 10 min at room temperature. To detect the degradation of the three ^68^Ga-labelled radiotracers, the supernatant was filtered through a water filter (0.22 μm) and analyzed by radio-HPLC.

Finally, in an EP tube coated with an anticoagulant, 0.2 mL of one of the three ^68^Ga-labelled radiotracers (37–74 MBq) was added to 1.0–1.4 mL of fresh human plasma collected from the left arm site. The mixture remained at room temperature. At 0, 1, 2, and 3 h after incubation, 0.2 mL of the above solution was added to acetonitrile (0.3 mL) to precipitate plasma proteins. Then, 300 µL of water was added, and the mixture was swirled. The plasma proteins were then separated by centrifugation at 12000 g for 10 min at room temperature. To detect the degradation of the three ^68^Ga-labelled radiotracers, the supernatant was filtered through a water filter (0.22 μm) and analysed by radio-HPLC.

The above experiments were repeated three times.

### Cell culture and tumor models

A detailed compilation of the SK-OV-3 and A549 cell growth is described in the supplemental materials.

Female BALB/c nude mice (6–8 weeks old) were utilized in all tumor-bearing mouse experiments. Twelve BALB/c nude mice were subcutaneously injected with SK-OV-3 cells (1 × 10^6^) into the right armpit. The mice were used for investigation after the tumor diameter reached approximately 0.5–1.1 cm.

### Western blot

For Western blotting analyses, the SK-OV-3 and A549 cell extracts were prepared by lysing cells with RIPA buffer with protease (P8340, Sigma Life Sciences) and phosphatase inhibitor cocktails 2 and 3 (P5726, P0044, Sigma Life Sciences). According to standard procedures, the supernatants of the lysates were collected for Western blotting after centrifugation at 12000 g (4 °C) for 15 min. The membranes were blocked with 3% BSA (bovine serum albumin) and then incubated with the primary antibodies PARP-1 (A9452; Cell Signaling Technologies) and β-actin anti-mouse (3700S, Cell Signaling Technologies). The signal was detected by a LiCor Odyssey CLx Imager (Lincoln, NE). ImageJ (version 1.8.0, National Institutes of Health, USA) was used for Western blot densitometry.

### Cell uptake

SK-OV-3 and A549 cells were seeded in 6-well plates (500 × 10^3^/well) for 24 h. Then, the cells were incubated separately with the three ^68^Ga-labelled radiotracers (740 kBq/1 mL in each well) at 37 °C. To eliminate unbound ^68^Ga-labelled radiotracers, the cells were rinsed three times with PBS (0.5 mL) 2 h after incubation. Cell suspensions were produced after adding pancreatin (0.5–0.8 mL). Finally, a γ-counter was used to measure the radioactivity. The radioactivity data are presented as CPM (counts per minute) with time-decay correction. Three replicate experiments were conducted.

### In vitrocell localization

SK-OV-3 cells were plated on a confocal petri dish (diameter = 60 mm) at 500 × 10^3^ cells/well density. After 24 h, the cells were incubated with a fluorescent compound FL-Olaparib (3-BODIPY-propanoic acid-conjugated Olaparib, 250 nM) alone for 1 h. The confocal dish was protected from light by wrapping it in tin foil. Three PBS washes (10 min each) were performed on each cell type. Paraformaldehyde was added for cell fixation, and DAPI was added for nuclear staining. The cells were rinsed twice with medium and once with PBS (5 min per wash) and observed using a confocal microscope (LEICA TCS SP5 II).

To determine the cellular localization of Olaparib and DOTA-Olaparib, FL-Olaparib was added to SK-OV-3 cells with an excess of competitive inhibitor (Olaparib, 25 mM; DOTA-Olaparib, 250 mM). After 1 h of incubation, to determine the uptake by SK-OV-3 cells, assays were carried out and repeated as stated above.

### Pharmacokinetics in normal mice

The pharmacokinetic parameters of the three ^68^Ga-labelled radiotracers were evaluated in normal mice. The three ^68^Ga-labelled radiotracers were injected into mice via the tail veins at a dose of 1.48–2.96 MBq/mouse in approximately 200 μL. Blood samples were obtained via the tail vein using tared capillary tubes at 1, 3, 5, 10, 15, 30, 60, 120, and 180 min after radiotracer injection. The blood volume drawn was approximately 0.5–0.8 dispense capillary volumes at each time point. Dispense capillary tube was from Shanghai Great Wall Scientific Instruments Store (size: 0.5 × 100; diameter: 0.5 mm; length: 100 mm). The radioactivity was determined with a γ-counter and decay-corrected to the injection time. All samples were weighed. The radioactive CPM (counts per minute) by dividing by the mass of blood in the capillary are presented as %ID/g. Finally, the pharmacokinetic parameters were analysed by GraphPad Prism 9.0.

### Plasma protein binding

To evaluate the plasma protein binding of the ^68^Ga-labelled radiotracers, mouse blood from the heart was centrifuged at 4000 g for 5 min. The upper layer of blood was transferred to an EP tube and stored overnight in a refrigerator at 4 °C.

One hundred microliters of the ^68^Ga-labelled radiotracer solution (0.74–1.48 MBq) was incubated with 100 μL of the above plasma. After 0.5, 1, and 2 h of incubation, the mixture was centrifuged at 15000 g for 10 min in a 30 K ultrafiltration tube. The radioactivity of the membrane and eluate was detected with a γ-counter. The radioactivity from the membrane, representing the tracer bound to plasma proteins, was estimated as a proportion of the total radioactivity of the sample. The experiment was repeated in triplicate.

### Ex vivobiodistribution in normal mice

Normal mice (6–8 weeks old, *n* = 5) were utilized in the biodistribution experiments. ^68^Ga-DOTA-Olaparib (0.74–2.96 MBq of 200 μL of saline) was administered via the lateral tail vein. The medication was allowed to circulate for 5, 15, 30, 60, 120, and 180 min before the mice were sacrificed. The radioactivity in the target tissues (blood, muscle, bone, liver, spleen, kidney, heart, lung, bladder, brain, small intestine, and stomach) was measured using a γ-counter. The organs were weighed, and the γ-counter activity readings were decay-corrected to the injection time. The corresponding tissue activity was then assessed using GraphPad Prism 9.0, and the data are presented as %ID/g using the following formula: [(activity in the target organ/target organ quality)/injected dose] * 100%.

### MicroPET/CT imaging

Six SK-OV-3 models were separated into two groups (^68^Ga-labelled radiotracers and blocked groups). The ^68^Ga-labelled radiotracers (2.96–5.55 MBq in 200 μL of saline) were administered via tail vein injection. Approximately 5 min before PET acquisition, the mice were anaesthetized by inhalation of a mixture of isoflurane and positioned in the scanner. The PET data for each mouse were acquired at 0.5, 1, and 2 h after injection. In the blocking studies, ^68^ Ga-DOTA-Olaparib was injected with a 500-fold excess of Olaparib (562.68 μg) in 200 μL of a solution consisting of 75% saline and 25% DMSO (dimethyl sulfoxide). PET acquisition was performed at 1 h postinjection. The target tissue’s radioactivity content was measured, and the tissue-associated activity was expressed as %ID/g.

### Ex vivo biodistribution (SK-OV-3 models and A549 models)

Biodistribution experiments were conducted with SK-OV-3 models (10–12 weeks old, *n* = 3). ^68^Ga-DOTA-Olaparib (1.85–5.55 MBq of ^68^Ga-DOTA-Olaparib in 200 μL of saline) was administered via the lateral tail vein. After allowing the medication to circulate for varying amounts of time (0.5, 1, and 2 h), three mice were sacrificed. The tissue radioactivity (tumor, blood, muscle, bone, liver, spleen, kidney, heart, lung, bladder, brain, small intestine, and stomach) was measured using a γ-counter. The organs were weighed, and the γ-counter activity readings were decay-corrected to the injection time. Finally, the associated tissue activity was calculated by GraphPad Prism 9.0, and the data are expressed as %ID/g.

To verify specific tumor accumulation, ^68^Ga-DOTA-Olaparib was administered with a 500-fold excess of Olaparib (530.94 μg) in 200 μL of a solution containing 75% saline and 25% DMSO via the lateral tail vein. After 1 h, mice (*n* = 3) were sacrificed to collect the tissues of interest. The associated tissue activity was measured as described above.

To further compare and validate the specific uptake of ^68^Ga-DOTA-Olaparib in different tumor models, biodistribution experiments were conducted with A549 models (10–12 weeks old, *n* = 3). ^68^Ga-DOTA-Olaparib (1.22–5.59 MBq of ^68^Ga-DOTA-Olaparib in 200 μL of saline) was administered via the lateral tail vein. After allowing the medication to circulate for varying amounts of time (0.5, 1, and 2 h), three mice were sacrificed. The tissue radioactivity (tumor, blood, muscle, bone, liver, spleen, kidney, heart, lung, bladder, brain, small intestine, and stomach) was measured using a γ-counter. The organs were weighed, and the γ-counter activity readings were decay-corrected to the injection time. Finally, the associated tissue activity was calculated by GraphPad Prism 9.0, and the data are expressed as %ID/g.

### Autoradiography and H&E staining

In the ex vivo biodistribution study, tumor tissues were excised and frozen at − 80 °C and divided into thin slices. To determine radiotracer distribution, consecutive sections were subjected to autoradiography and H&E staining (haematoxylin and eosin staining) for morphologic characterization of tissue pathology. For autoradiography, portions of the slices were placed on an image plate. To visualize the radioactive signal, the plate was scanned and read following 2.5 h of exposure. The remaining sections were subjected to immunohistochemistry.

### Immunohistochemistry

The protein PARP-1 expression immunohistochemistry staining was conducted according to the previous protocol [34]. The above SK-OV-3 tumor slides were then incubated with the recombinant anti-PARP-1 antibody (1:200 dilution, ab191217, Abcam).

### Statistical analysis

All experimental results are presented as the mean ± SEM (standard error of the mean). GraphPad Prism (version 9.0, San Diego, USA) was utilized to conduct unpaired *t*-tests to identify significant differences. *P* values < 0.05 were considered statistically significant.

## Results

### Design of the precursors and PARP-1 binding mode analysis

**Fig. 1:**
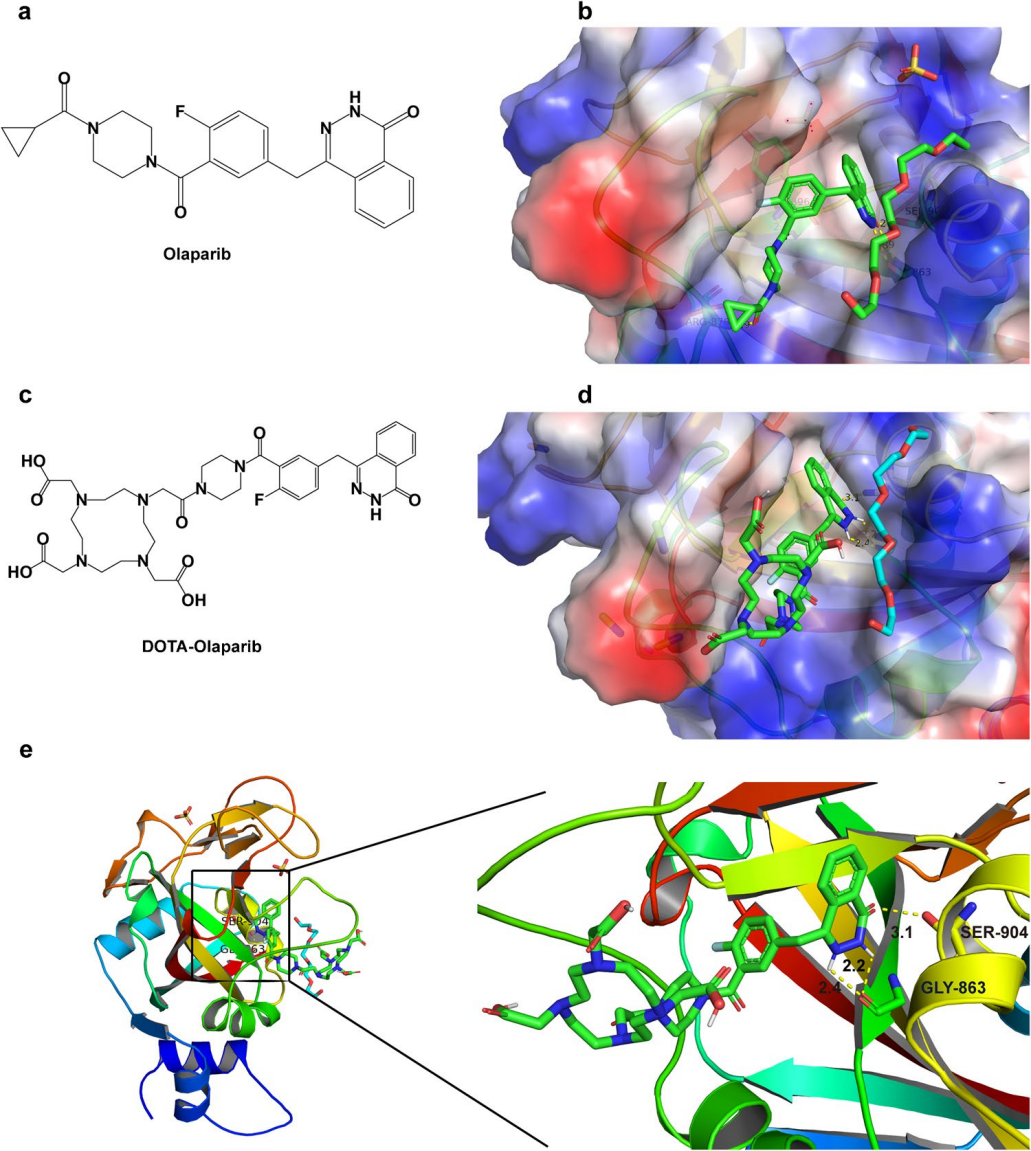
Design of ^68^ Ga-DOTA-Olaparib for PARP-1 binding. (**a**) Chemical structure of the PARP-1 inhibitor Olaparib. (**b**) Co-crystal structure of Olaparib bound to the PARP-1 catalytic domain (PDB ID: 5DS3). (**c**) Chemical structure of DOTA-Olaparib. (**d**) Molecular docking of DOTA-Olaparib to PARP-1. (**e**) H-bonding interactions between DOTA-Olaparib and PARP-1 residues

To find a potential PARP-targeted radioactive imaging probe, Olaparib was chosen as the PARP ligand due to the excellent activity against PARP (Fig. 1a) [35]. The co-crystal structure of Olaparib in conjunction with PARP-1 (Fig. 1b) indicated that its amide group on Olaparib was critical for binding [36]. The cyclopropyl ring of Olaparib was situated near the entrance of the ligand–protein binding pocket, constituting a potential place for further modification. The three PARP-targeted radiopharmaceuticals were designed and synthesized based on the above design principles. The cyclopropyl ring of Olaparib was replaced by DOTA for ^68^Ga labelling.

The DOTA-Olaparib molecular docking results are shown in Fig. 1d–e. The original binding pocket and hydrogen bonds between the amide moiety of Olaparib and PARP-1 were maintained. In addition, it was observed that the terminal electrophilic amide group could effectively approach PARP-1 residues GLY863 and SER904, indicating that the PARP-1 protein might form a stable hydrogen bond with the amide group of DOTA-Olaparib. These results suggested the great promise of DOTA-Olaparib as a specific molecule due to its excellent interactions with the binding pocket of PARP-1. To investigate the SAR to find the optimal structure for PARP-1 binding in vivo, we designed two other precursors based on Olaparib and tried to increase lipid solubility by introducing a fatty chain and adding amide bonds. Similarly, we performed computer simulations, and the potential binding modes of DOTA-GABA-Olaparib and DOTA-(Gly)_3_-Olaparib with PARP-1 are shown in Fig. S5. The results indicated that these three precursors entered the same binding pocket as Olaparib.

### Synthetic chemistry

The compound 4-(4-fluoro-3-(piperazine-1-carbonyl)benzyl)phthalazin-1(2H)-one **(compound 1)** was used as the starting material for Olaparib-based radiopharmaceutical synthesis. As shown in Fig. S1, DOTA-Olaparib was obtained in a two-step process. In high yield, Starting compound **1** was reacted with the protected DOTA group and concentrated hydrochloric acid. To investigate the effects of diverse linker lengths and the number of amide bonds on drug activity, the function of the amide derivatives was explored (Fig. S2-S3). Compounds DOTA-GABA-Olaparib and DOTA-(Gly)_3_-Olaparib were obtained in excellent yields by the reaction of compound **1** with Boc-γ-ABU-OH **(4)** and Boc-(Gly)_3_-OH **(8)**, respectively. To give different intermediates, the amidation reactions between compound **1** and different acids proceeded smoothly in the presence of the catalytic agent HATU (2-(7-Azabenzotriazol-1-yl)-N,N,N',N'-tetramethyluronium hexafluorophosphate) via nucleophilic substitution reactions. The intermediates were directly reacted with 6 N aq. HCl to obtain the radiopharmaceutical precursors. These new precursors synthesized were characterized spectroscopically before proceeding with radioisotope labelling (Fig. S12-S30).

### Radiochemistry

**Fig. 2:**
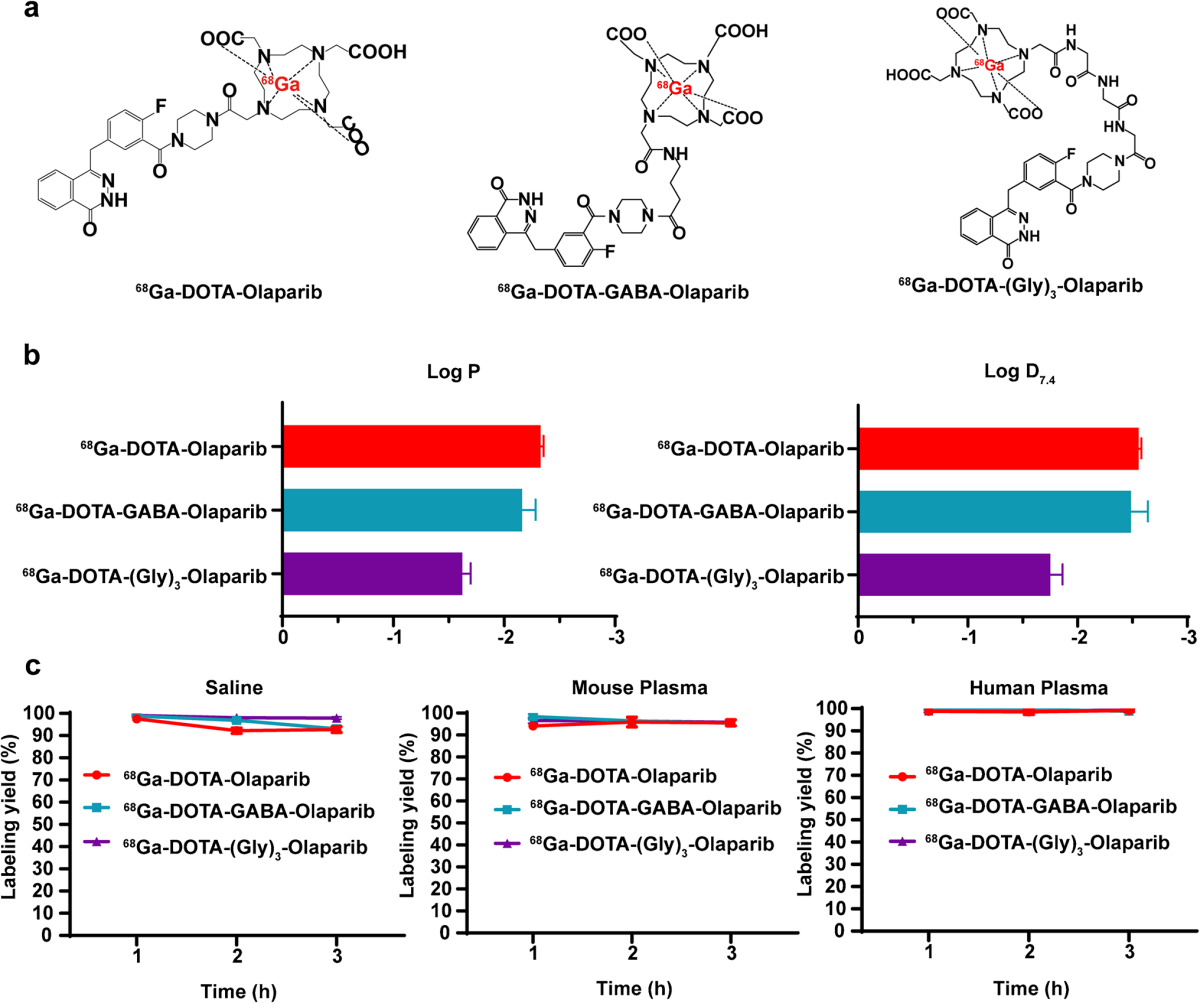
Chemical structures, solubility, and stability of the ^68^Ga-labelled radiotracers. (**a**) Chemical structures of the three ^68^Ga-labelled radiotracers. (**b**) The log P_octanol/water_ and log D_octanol/PBS_ values for three ^68^Ga-labelled radiotracers. (**c**) Stability of the three.^68^Ga-labelled radiotracers in saline (0, 1, 2, and 3 h), mouse plasma (0, 1, 2, and 3 h), and human plasma (0, 1, 2, and 3 h)

The chemical structures of the three ^68^Ga-labelled radiotracers are shown in Fig. 2a. The RCP (radiochemical purity) of each was > 97%. The distance the ^68^Ga-labelled radiotracers travelled on radio-TLC was 70–110 mm, and that of ^68^GaCl_3_ was 40–60 mm. Furthermore, the high RCP (> 97%) of ^68^Ga-labelled radiotracers was confirmed by radio-HPLC. The retention times of the three precursors (UV peak: DOTA-Olaparib, 8.211 min; DOTA-GABA-Olaparib, 7.787 min; DOTA-(Gly)_3_-Olaparib, 6.949 min) and ^68^Ga-labelled radiotracers (radioactive peak) were between approximately 6–8 min (^68^Ga-DOTA-Olaparib, 8.523 min; ^68^Ga-DOTA-GABA-Olaparib, 8.099 min; ^68^Ga-DOTA-(Gly)_3_-Olaparib, 7.648 min). These similar retention times indicated that the precursors had been successfully radiolabelled with gallium-68 (Fig. S6a-b).

### Binding affinity

The binding affinity of DOTA-Olaparib, DOTA-GABA-Olaparib, and DOTA-(Gly)_3_-Olaparib for PARP-1 was determined using SPR imaging, the results of which are shown in Fig. S7a-c. The Kd values for compound DOTA-Olaparib, DOTA-GABA-Olaparib, and DOTA-(Gly)_3_-Olaparib were 14.46 nM, 1.10 nM, and 3.58 nM, respectively. These results indicate that the three radiotracers have a high affinity towards PARP-1.

### Partition coefficients of the three ^68^Ga-labelled radiotracers

The hydrophilicity of the ^68^Ga-labelled radiotracers was evaluated based on the proportional distribution between aqueous (ultrapure water or PBS) and organic (1-octanol) phases. The log P_octanol/water_ and log D_octanol/PBS_ values of ^68^Ga-DOTA-Olaparib were -2.33 ± 0.028 and -2.56 ± 0.026, respectively. The partition coefficients of ^68^Ga-DOTA-GABA-Olaparib were -2.16 ± 0.12 (log P_octanol/water_) and -2.49 ± 0.15 (log D_octanol/PBS_). The partition coefficients were -1.62 ± 0.075 (log P_octanol/water_) and -1.75 ± 0.11 (log D_octanol/PBS_) for ^68^Ga-DOTA-(Gly)_3_-Olaparib. Figure 2b shows that ^68^Ga-DOTA-Olaparib had the strongest hydrophilicity among the three radiopharmaceuticals.

### Stability of the ^68^Ga-labelled radiotracers

First, stability studies of the ^68^Ga-labelled radiotracers were performed in saline for 3 h. As shown in Fig. S8a, greater than 90% of the prototype radiotracers remained in saline after 3 h of incubation, and no degradation was observed. To further simulate the complicated internal environment, the stability of the ^68^Ga-labelled radiotracers was evaluated in mouse plasma and human plasma. The results are illustrated in Fig. S8b-c, more than 90% of the ^68^Ga-labelled radiotracers remained in fresh mouse plasma and human plasma (Fig. 2c).

### High cell uptake of the three ^68^Ga-labelled radiotracers

**Fig. 3:**
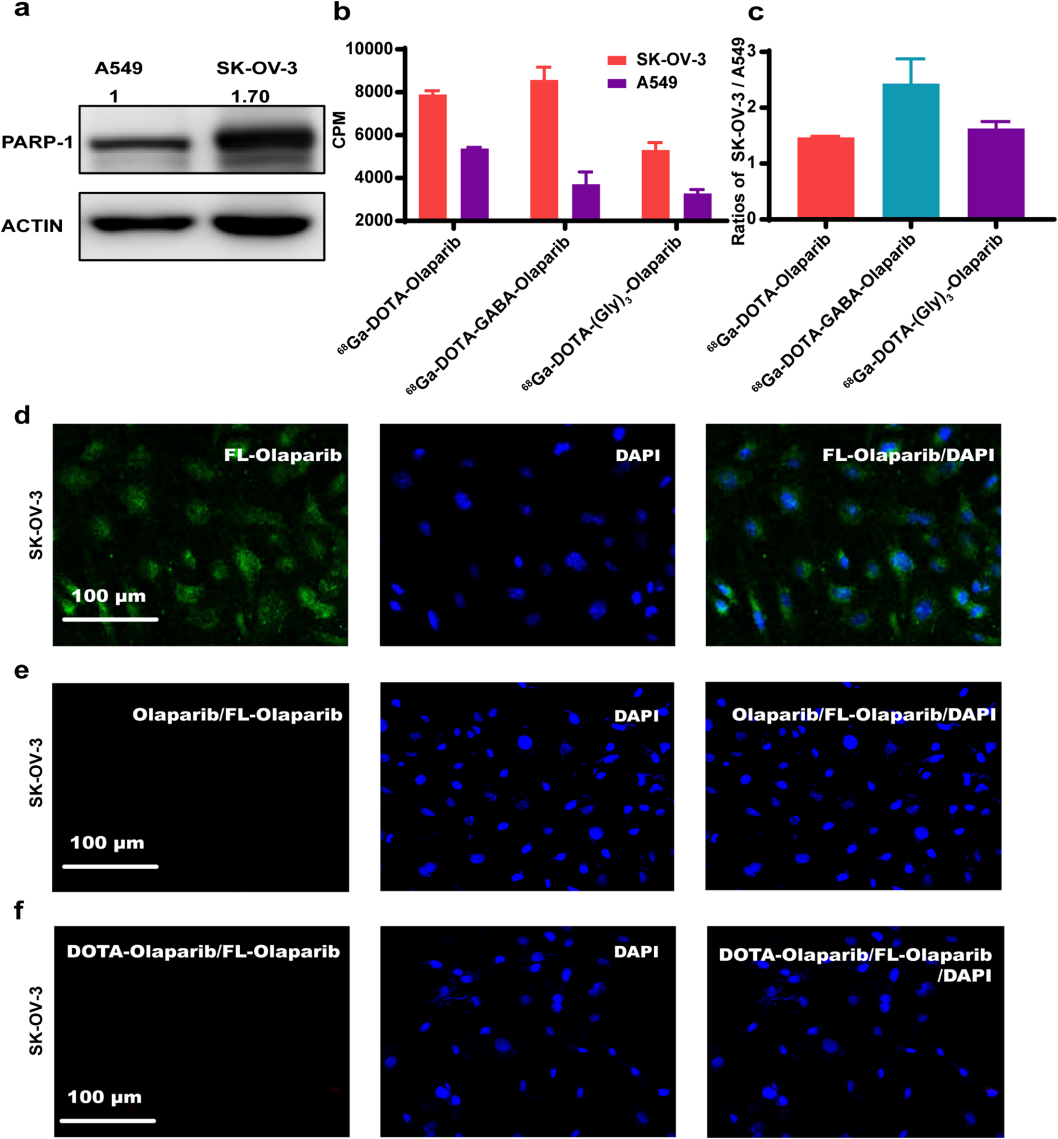
Cell uptake of the three ^68^Ga-labelled radiotracers. (**a**) Western blot analysis probing the expression of PARP-1 in A549 and SK-OV-3 cells. (**b**) Cell uptake of the ^68^Ga-labelled radiotracers in SK-OV-3 and A549 cells after 2 h of incubation. (**c**) The SK-OV-3/A549 uptake ratios of the.^68^Ga-labelled radiotracers. (**d**) Confocal images of cells stained with FL-Olaparib (green, left) and DAPI (blue, middle) alone. Confocal image of cells stained with FL-Olaparib and DAPI (right). (**e**) Confocal images of cells stained with FL-Olaparib with a 100-fold excess of Olaparib (left) and DAPI (blue, middle) alone. Confocal image of cells stained with FL-Olaparib with DAPI and a 100-fold excess of Olaparib (right). (**f**) Confocal images of cells stained with FL-Olaparib with a 1000-fold excess of DOTA-Olaparib (left) and DAPI (blue, middle) alone. Confocal image of cells stained with FL-Olaparib with DAPI and a 1000-fold excess of DOTA-Olaparib (right)

PARP-1 expression in SK-OV-3 and A549 cells was determined by Western blotting. The results showed high PARP-1 expression in SK-OV-3 cells and low PARP-1 expression in A549 cells (Fig. 3a).

Because of the elevated levels of PARP-1 in SK-OV-3 cells, the uptake of the three ^68^Ga-labelled radiotracers was significantly higher in SK-OV-3 cells than in A549 cells (Fig. 3b). The ^68^Ga-DOTA-Olaparib, ^68^Ga-DOTA-GABA-Olaparib, and ^68^Ga-DOTA-(Gly)_3_-Olaparib of SK-OV-3/A549 uptake ratios were 1.47 ± 0.019, 2.43 ± 0.45, and 1.63 ± 0.13, respectively (Fig. 3c). The above results agree with the relative PARP-1 expression levels in both cell types (SK-OV-3/A549 = 1.70) observed by Western blotting.

### Localization of intracellular fluorescence determined by ^68^Ga-labelled radiotracer imaging

To demonstrate the intracellular localization between the ^68^Ga-labelled radiotracers and PARP-1 in vitro, we chose the fluorescence imaging agent FL-Olaparib (the structure is shown in Fig. S4). After incubation with FL-Olaparib, the strong green fluorescence signals in SK-OV-3 cells were colocalized with the signals from the cytoplasm and nucleus (blue) in cells where PARP-1 was expressed (Fig. 3d) [37].

Because the three precursors bound in the same PARP-1 binding pocket as Olaparib, we chose ^68^Ga-DOTA-Olaparib to examine the intracellular localization of the ^68^Ga-labelled radiotracers in vitro*.* By separately presaturation with a molar excess of Olaparib (100-fold) and DOTA-Olaparib (1000-fold), a substantial reduction in fluorescence signals was observed (Fig. 3e–f). These results demonstrated that the ^68^Ga-labelled radiotracers could be located intracellularly where PARP-1 is expressed.

### Pharmacokinetics and plasma protein binding of ^68^Ga-labelled radiotracers

**Fig. 4:**
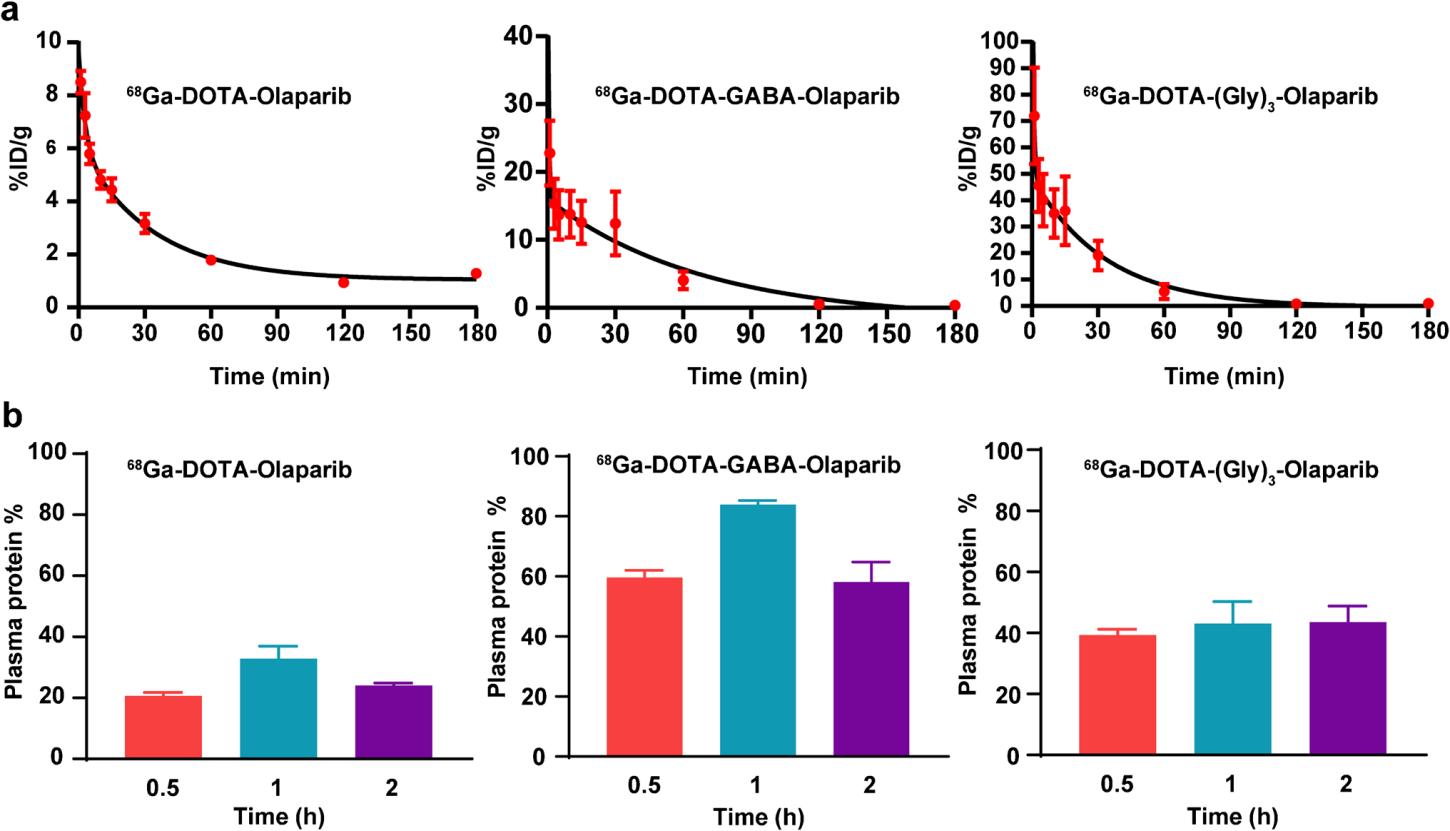
Pharmacokinetics of the three ^68^Ga-labelled radiotracers. (**a**) Blood clearance of the three ^68^Ga-labelled radiotracers in vivo. (**b**) Plasma protein binding of the three ^68^Ga-labelled radiotracers at 0.5, 1, and 2 h

To investigate the blood clearance of the ^68^Ga-labelled radiotracers, the pharmacokinetic parameters of the three ^68^Ga-labelled radiotracers were determined in normal mice (Fig. 4a). Unlike Olaparib, the radioactivity in mouse blood indicated that the three ^68^Ga-labelled radiotracers were eliminated reasonably rapidly in vivo. The distribution-phase half-life (t_1/2α_) values of the three ^68^Ga-labelled radiotracers were between approximately 0–2 min (^68^Ga-DOTA-Olaparib, 1.97 min; ^68^Ga-DOTA-GABA-Olaparib, 0.31 min; ^68^Ga-DOTA-(Gly)_3_-Olaparib, 0.40 min). The clear-phase half-life (t_1/2β_) values of the three ^68^Ga-labelled radiotracers were between approximately 20–50 min (^68^Ga-DOTA-Olaparib, 22.82 min; ^68^Ga-DOTA-GABA-Olaparib, 47.69 min; ^68^Ga-DOTA-(Gly)_3_-Olaparib, 23.19 min).

The mouse blood plasma protein binding of ^68^Ga-DOTA-Olaparib was 20.59 ± 1.23% at 0.5 h, 32.85 ± 4.11% at 1 h, and 24.04 ± 0.80% at 2 h. These binding results were significantly lower than those of ^68^Ga-DOTA-GABA-Olaparib (59.62 ± 2.40% at 0.5 h, 83.84 ± 1.42% at 1 h, and 58.13 ± 6.63% at 2 h) and ^68^Ga-DOTA-(Gly)_3_-Olaparib (39.32 ± 1.89% at 0.5 h, 43.08 ± 7.20% at 1 h, and 43.56 ± 5.24% at 2 h). The results demonstrated that ^68^Ga-DOTA-Olaparib presented significantly lower plasma protein binding and more rapid clearance.

### MicroPET/CT imaging

**Fig. 5:**
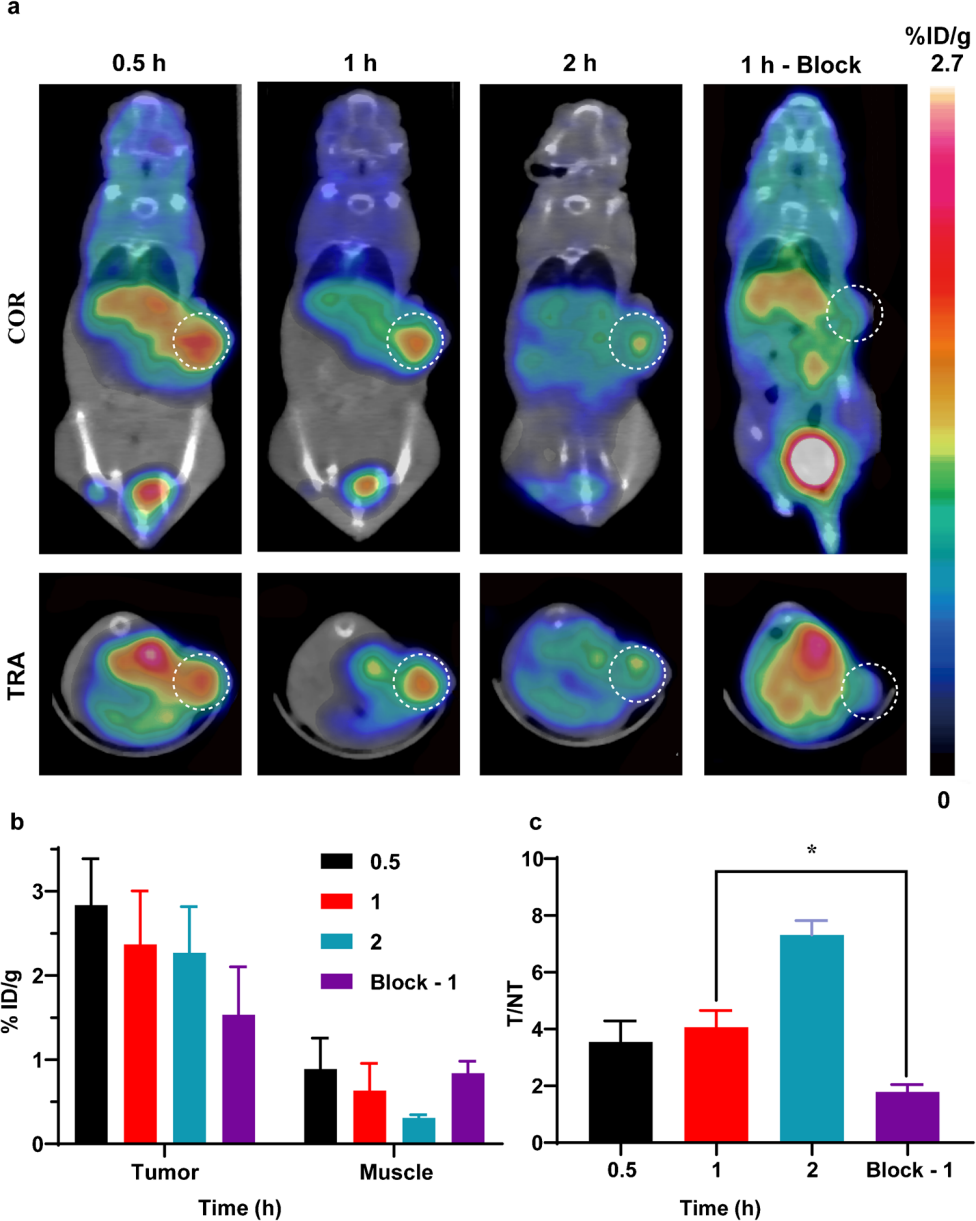
PET imaging of ^68^Ga-DOTA-Olaparib. (**a**) MicroPET/CT images of.^68^Ga-DOTA-Olaparib in SK-OV-3 models at 0.5, 1, and 2 h after administration. (**b**) Quantification of the regions of interest (ROIs) in SK-OV-3 models expressed as %ID/g. (**c**) T/M (tumor-to-muscle) ratios (*P = 0.0238 < 0.05, *n* = 3)

^68^Ga-DOTA-Olaparib PET/CT imaging showed a significant concentration of radioactivity in the SK-OV-3 tumors (Fig. 5a). The uptake of radioactive ^68^Ga-DOTA-Olaparib in the SK-OV-3 models was approximately 2.83 ± 0.32%ID/g (0.5 h), 2.37 ± 0.37%ID/g (1 h), and 2.27 ± 0.32%ID/g (2 h) as determined by quantitative calculations from the PET images (Fig. 5b). The T/M ratios of ^68^Ga-DOTA-Olaparib were approximately 3.54 ± 0.74 (0.5 h), 4.07 ± 0.59 (1 h), and 7.31 ± 0.51 (2 h).

In the blocking experiment, there was a significant difference in the T/M ratios between the unblocked group and the group blocked with a 500-fold excess of Olaparib (Fig. 5c, 4.07 ± 0.59 vs. 1.79 ± 0.26 at 1 h). This result confirmed the specificity of ^68^Ga-DOTA-Olaparib for PARP.

By comparison, the tumor uptake of ^68^Ga-DOTA-GABA-Olaparib (0.96 ± 0.14%ID/g at 0.5 h; 0.56 ± 0.12%ID/g at 1 h; 0.98 ± 0.53%ID/g at 2 h) and ^68^Ga-DOTA-(Gly)_3_-Olaparib (0.96 ± 0.13%ID/g at 0.5 h; 0.57 ± 0.068%ID/g at 1 h; 0.91 ± 0.11%ID/g at 2 h) was much lower than that of ^68^Ga-DOTA-Olaparib. The PET/CT images are displayed in the supplemental materials (Fig. S9-S10).

### Ex vivo biodistribution (SK-OV-3 models, A549 models, and normal mice)

**Fig. 6:**
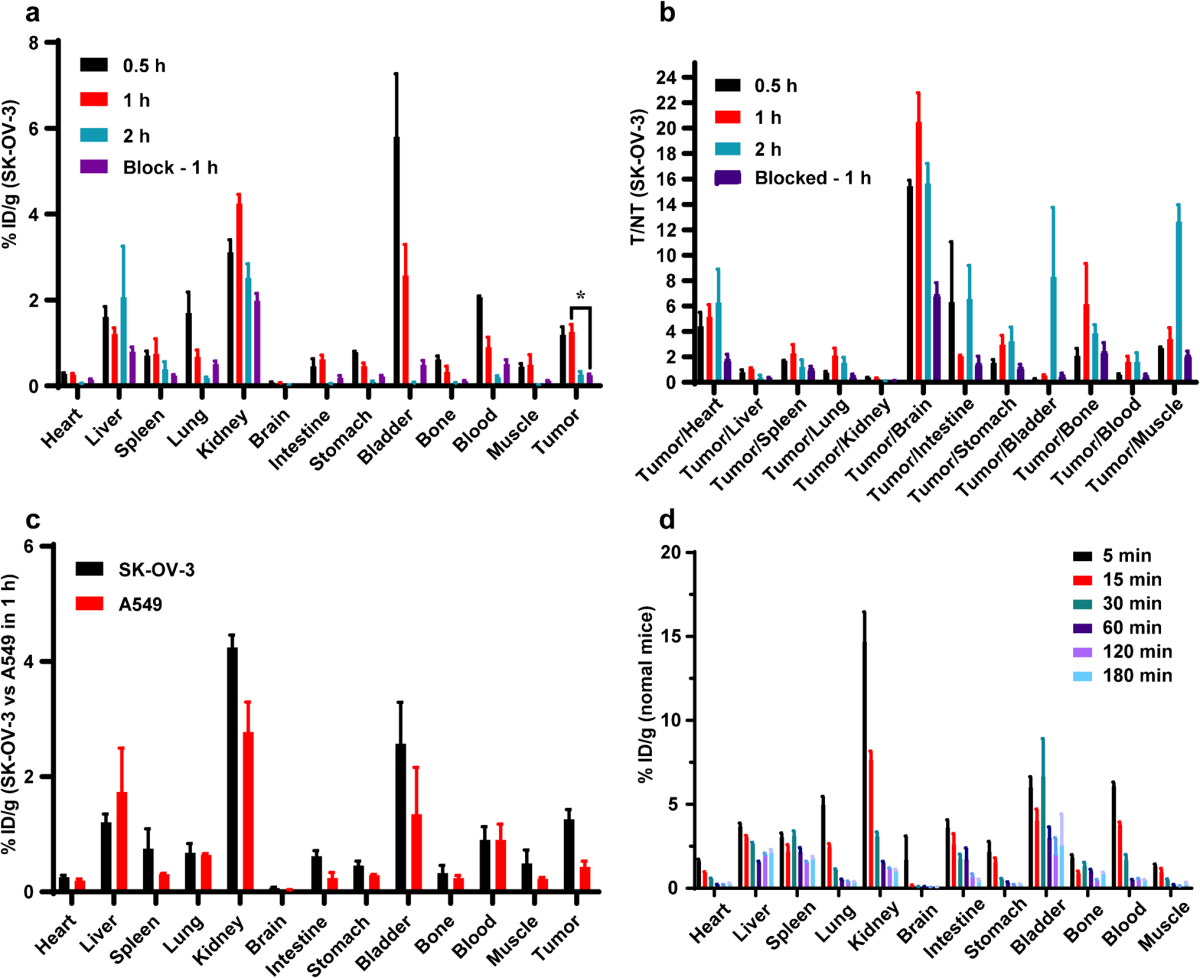
In vivo efficacy and specificity of ^68^Ga-DOTA-Olaparib. (**a**) Biodistribution of ^68^Ga-DOTA-Olaparib in SK-OV-3 models (*P = 0.046 < 0.05, *n* = 3). (**b**) The ratios of tumor-to-nontarget tissue (*n* = 3). (**c**) Biodistribution of ^68^Ga-DOTA-Olaparib in SK-OV-3 and A549 tumor-bearing rats, respectively, at 1 h post-injection time point (*n* = 3). (**d**) Biodistribution of ^68^Ga-DOTA-Olaparib in normal mice (*n* = 5)

The efficacy of ^68^Ga-DOTA-Olaparib to target tumors in vivo was further assessed via an ex vivo biodistribution investigation (Fig. 6a). At 1 h postinjection, there was a significant accumulation of radioactivity (1.26 ± 0.17%ID/g) in the tumors (Fig. 6b). When a 500-fold excess of Olaparib was administered to the mice, the accumulation of ^68^Ga-DOTA-Olaparib in the tumors dropped to 0.25 ± 0.027%ID/g.

To further compare and validate the specific uptake of ^68^Ga-DOTA-Olaparib in different tumor models, we further selected the A549 models with lower PARP-1 expression for the ^68^Ga-DOTA-Olaparib biodistribution investigation (Figure. S10a-b). As shown in Fig. 6c, ^68^Ga-DOTA-Olaparib had significantly lower tumor uptake in the A549 models (0.43 ± 0.10%ID/g at 1 h) compared to the higher tumor-specific uptake of the probe in the SK-OV-3 models (1.26 ± 0.17%ID/g at 1 h). The ex vivo biodistribution and PET imaging in SK-OV-3 model results demonstrated that ^68^Ga-DOTA-Olaparib was excreted via the urinary system. A high accumulation of ^68^Ga-DOTA-Olaparib was visualized in the kidneys (3.11 ± 0.29%ID/g at 0.5 h, 4.24 ± 0.22%ID/g at 1 h, and 2.51 ± 0.33%ID/g at 2 h) and bladder (5.80 ± 1.47%ID/g at 0.5 h, 2.57 ± 0.72%ID/g at 1 h, and 0.064 ± 0.025%ID/g at 2 h). At the same time, there was a relatively low distribution of radioactivity in other tissues at 0.5, 1, and 2 h after administration.

Additionally, the biodistribution investigation revealed remarkable T/M (12.64 ± 1.32), tumor-to-brain (15.63 ± 1.58), and tumor-to-bone (3.85 ± 0.68) ratios at 2 h (Fig. 6c).

It was then necessary to further understand the ADME (absorption, distribution, metabolism, and excretion) of the drug in the different body organ tissues for a longer time after injection. According to the biodistribution in normal mice, ^68^Ga-DOTA-Olaparib was cleared from the body in the order of kidney-bladder-urine. In addition, the radioactivity was eliminated quickly, with most organs showing little radioactivity 1–2 h after injection. (Fig. 6d).

### Autoradiography, H&E staining, and immunohistochemistry

**Fig. 7:**
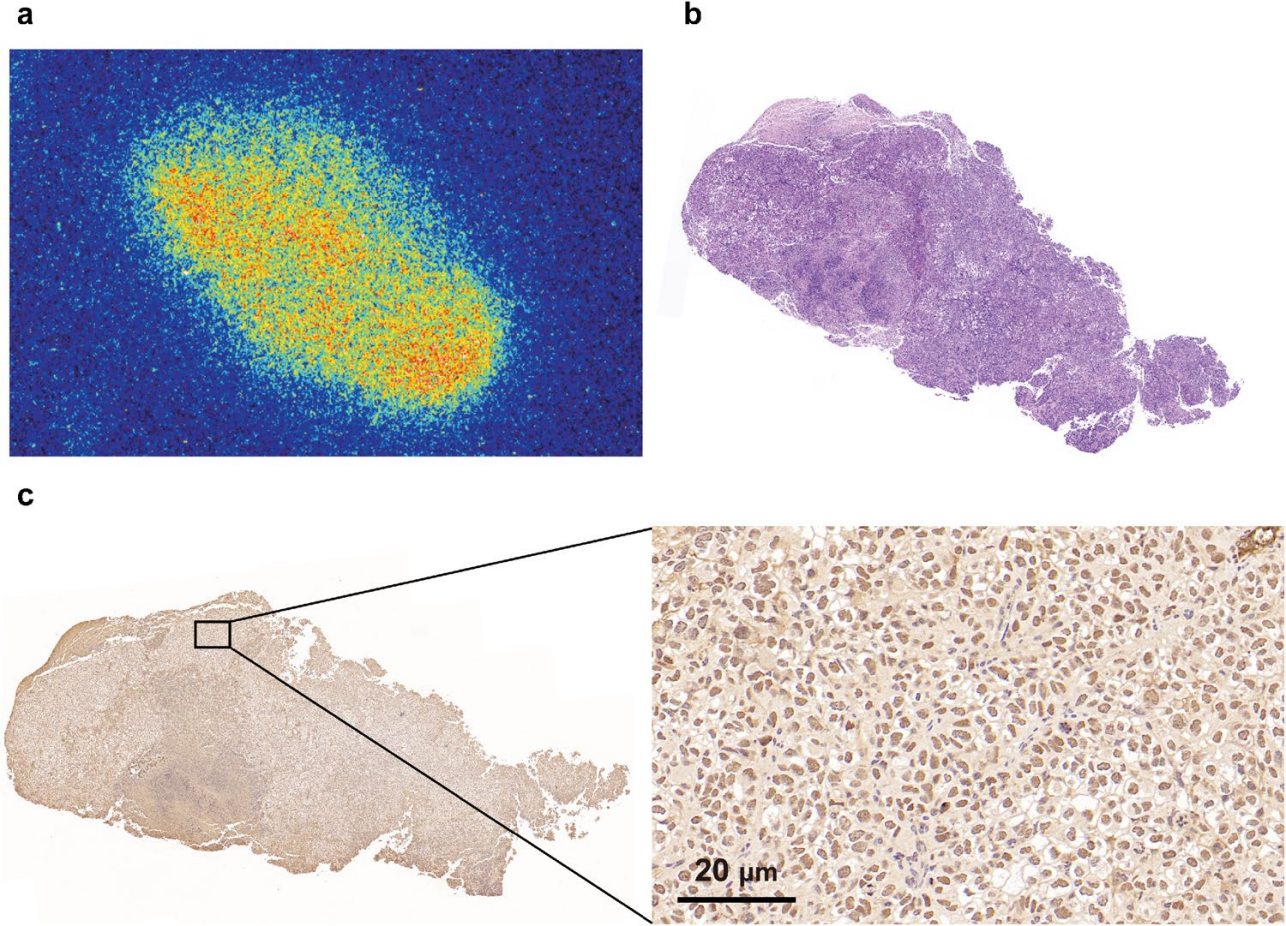
Association of ^68^Ga-DOTA-Olaparib signalling with PARP-1 expression at the tumor tissue level. (**a**) Autoradiography and (**b**) H&E staining of SK-OV-3 models injected with ^68^Ga-DOTA-Olaparib. (**c**) Representative image showing PARP-1 immunohistochemical staining

To further investigate the correlation between the distribution of radioactivity and PARP-1 expression, adjacent slices were subjected to autoradiography with ^68^Ga-DOTA-Olaparib and stained. The ^68^Ga-DOTA-Olaparib signal was noticeably concentrated in the tumors (Fig. 7a), comparable to the H&E staining experiment performed in parallel (Fig. 7b).

To detect the expression of PARP-1 in SK-OV-3 xenograft tissue and its localization in the cells (Fig. 7c), the immunohistochemistry staining of the tumor tissue revealed a high expression level of PARP-1 in SK-OV-3 xenograft tissue. As expected, PARP-1 was mainly expressed in the nucleus. Moreover, the PARP-1 immunohistochemistry staining indicated comparably high PARP-1 expression in SK-OV-3 xenograft tissue, confirming the autoradiography findings’ validity.

## Discussion

Since imaging with ^68^Ga-labelled nuclides has not been reported in the field of specific PARP-targeted probes to date (to our knowledge), we demonstrated the first investigation of ^68^Ga-PARP imaging agents in vivo based on Olaparib, the FDA-approved PARP inhibitor [38]. Intensive research is being conducted on PARP inhibitors, yet many clinical trials have been developed to evaluate the effects of different drugs in synthetic lethal combinations. To measure PARP expression in tumors and monitor patient responses to PARP therapy, imaging the PARP enzyme family using radioactive probes has been proposed [16].

Transforming PARP inhibitors into imaging probes with well-established tumor imaging modalities will face many challenges. The common characteristics of most inhibitors are poor water solubility [20], a relatively long mean terminal half-life (6.10 h following a single oral dose in patients with cancer) [39], and excessive plasma protein binding (75.9%) [20]. The current PARP radiotracers rapidly accumulate in the liver and are excreted via bile, which is suboptimal for imaging abdominal lesions (liver, intestines, stomach, and ovaries). This physiological distribution may affect lesion detection significantly since the abdomen is the common metastatic site of breast and ovarian cancer. The application of ^18^F-labelled PARP inhibitors to evaluate PARP in both cancer types would therefore be subject to certain restrictions.

To develop PARP radiotracers suitable for abdominal imaging, we successfully designed Olaparib derivatives through computer-aided drug design. No significant altering of the binding mode to PARP while improving the pharmacokinetic properties of Olaparib. Then, the Olaparib derivatives were synthesized, radiolabelled, and evaluated in a PARP-1-positive tumor model. Compared with the existing PARP probes with poor water solubility and excessive accumulation in the liver and intestine, ^68^Ga-DOTA-Olaparib is a radioactive PARP-specific targeting probe with good water solubility. It was rapidly distributed within the body in 1.97 min and dramatically enhanced the in vivo clearance rate with significantly lower plasma protein binding (24.04 ± 0.80% at 2 h). In contrast to previous PARP probes, after a single bolus intravenous injection, the blood half-life of ^68^Ga-DOTA-Olaparib exhibited rapid pharmacokinetics and rapid clearance in vivo over the first 22.82 min. The route of excretion was successfully shifted toward renal clearance. In contrast to the kidney, the abdomen had significantly lower radioactive signal retention. ^68^Ga-DOTA-Olaparib was rapidly cleared from the non-target organs, leading to a substantial rise in the tumor-to-nontarget organ ratios 1 h after administration (tumor/intestine: 2.05 ± 0.072 and tumor/stomach: 2.97 ± 0.73).

Consistent with PARP-1 activation in ovarian tumors, ^68^Ga-DOTA-Olaparib PET/CT imaging displayed high tumor accumulation in the SK-OV-3 model. Furthermore, the high tumor accumulation of ^68^Ga-DOTA-Olaparib could be blocked by excessive Olaparib, indicating that the tumor accumulation of ^68^Ga-DOTA-Olaparib was PARP specific. There was a significant difference in the T/M ratio between the unblocked and blocked groups. The high accumulation of ^68^Ga-DOTA-Olaparib in the tumor was further validated by autoradiography of tumor slices, which revealed the preferential distribution of the ^68^Ga-DOTA-Olaparib in the tumor areas, which was confirmed by histology. Immunohistochemistry staining of tumor tissue revealed a high expression level of PARP-1 in SK-OV-3 xenograft tissue. Moreover, PARP-1 was mainly expressed in the nucleus at the cellular level. FL-Olaparib induced robust fluorescence that was retainable by PARP-1. The decrease in the fluorescence signal in the presence of DOTA-Olaparib verified the efficacy of ^68^Ga-DOTA-Olaparib in diffusing into the cell nucleus to bind to PARP-1.

It should be noted that there were some limitations in the present study. Notably, the tumor retention time of the ^68^Ga-labelled radiotracers was relatively short. On the other hand, in contrast to ^18^F imaging agents, the %ID/g of ^68^Ga-DOTA-Olaparib in tumors was lower in the ex vivo biodistribution study. These problems still require improvement. Finally, the increased molecular weight and decreased lipid solubility of the ^68^Ga-labelled radiotracers will reduce their ability to cross cell membranes and bind to PARP. We are currently working on solving these problems.

## Conclusion

In the present study, we designed, synthesized, and profiled a series of ^68^Ga-labelled radiotracers based on Olaparib for PARP-targeted imaging. As the first ^68^Ga-labelled PARP inhibitors, the radiotracers presented here can be produced by convenient manual operation in high radiolabelling yield. Because of these promising results, including the rapid in vivo clearance and high contrast imaging in mouse models, we anticipate that ^68^ Ga-DOTA-Olaparib may be a promising radiotracer for monitoring ovarian cancer tissues with elevated PARP expression and detecting abdominal tumor metastases.

## Supplementary Information

Below is the link to the electronic supplementary material.

Supplementary file1 (PDF 7876 KB)

## Data Availability

The original data are available upon request.

## References

[1] Russo G, Tramontano A, Iodice I, Chiariotti L, Pezone A (2021). Epigenome Chaos: Stochastic and Deterministic DNA Methylation Events Drive Cancer Evolution. Cancers.

[2] Tubbs A, Nussenzweig A (2017). Endogenous DNA Damage as a Source of Genomic Instability in Cancer. Cell.

[3] Drew Y, Plummer R (2009). PARP inhibitors in cancer therapy: Two modes of attack on the cancer cell widening the clinical applications. Drug Resist Update.

[4] Zhao Y, Zhang LX, Jiang T, Long J, Ma ZY, Lu AP (2020). The ups and downs of Poly(ADP-ribose) Polymerase-1 inhibitors in cancer therapy-Current progress and future direction. Eur J Med Chem.

[5] Makvandi M, Lee H, Puentes LN, Reilly SW, Rathi KS, Weng CC (2019). Targeting PARP-1 with Alpha-Particles Is Potently Cytotoxic to Human Neuroblastoma in Preclinical Models. Mol Cancer Ther.

[6] Wang YJ, Luo WB, Wang YF (2019). PARP-1 and its associated nucleases in DNA damage response. DNA Repair.

[7] Zandarashvili L, Langelier MF, Velagapudi UK, Hancock MA, Steffen JD, Billur R (2020). Structural basis for allosteric PARP-1 retention on DNA breaks. Science.

[8] Vaitsiankova A, Burdova K, Sobol M, Gautam A, Benada O, Hanzlikova H (2022). PARP inhibition impedes the maturation of nascent DNA strands during DNA replication. Nat Struct Mol Biol.

[9] Chalmers AJ, Lakshman M, Chan N, Bristow RG (2010). Poly(ADP-Ribose) Polymerase Inhibition as a Model for Synthetic Lethality in Developing Radiation Oncology Targets. Semin Radiat Oncol.

[10] Leung M, Rosen D, Fields S, Cesano A, Budman DR (2011). Poly(ADP-Ribose) Polymerase-1 Inhibition: Preclinical and Clinical Development of Synthetic Lethality. Mol Med.

[11] Kim DS, Camacho CV, Nagari A, Malladi VS, Challa S, Kraus WL (2019). Activation of PARP-1 by snoRNAs Controls Ribosome Biogenesis and Cell Growth via the RNA Helicase DDX21. Mol Cell.

[12] Kim DS, Camacho CV, Kraus WL (2021). Alternate therapeutic pathways for PARP inhibitors and potential mechanisms of resistance. Exp Mol Med.

[13] Scott LJ (2017). Niraparib: First Global Approval. Drugs.

[14] Thomas A, Murai J, Pommier Y (2018). The evolving landscape of predictive biomarkers of response to PARP inhibitors. J Clin Invest.

[15] Edmonds CE, Makvandi M, Lieberman BP, Xu K, Zeng C, Li S (2016). (18)F FluorThanatrace uptake as a marker of PARP1 expression and activity in breast cancer. Am J Nucl Med Mol Imaging.

[16] Wilson TC, Xavier MA, Knight J, Verhoog S, Torres JB, Mosley M (2019). PET Imaging of PARP Expression Using F-18-Olaparib. J Nucl Med.

[17] Reiner T, Lacy J, Keliher EJ, Yang KS, Ullal A, Kohler RH (2012). Imaging therapeutic PARP inhibition in vivo through bioorthogonally developed companion imaging agents. Neoplasia.

[18] Carlucci G, Carney B, Brand C, Kossatz S, Irwin CP, Carlin SD (2015). Dual-Modality Optical/PET Imaging of PARP1 in Glioblastoma. Mol Imaging Biol.

[19] Carney B, Carlucci G, Salinas B, Di Gialleonardo V, Kossatz S, Vansteene A (2016). Non-invasive PET Imaging of PARP1 Expression in Glioblastoma Models. Mol Imaging Biol.

[20] Zmuda F, Blair A, Liuzzi MC, Malviya G, Chalmers AJ, Lewis D (2018). An (18)F-Labeled Poly(ADP-ribose) Polymerase Positron Emission Tomography Imaging Agent. J Med Chem.

[21] Reilly SW, Puentes LN, Schmitz A, Hsieh CJ, Weng CC, Hou C (2019). Synthesis and evaluation of an AZD2461 [(18)F]PET probe in non-human primates reveals the PARP-1 inhibitor to be non-blood-brain barrier penetrant. Bioorg Chem.

[22] Guibbal F, Hopkins SL, Pacelli A, Isenegger PG, Mosley M, Torres JB (2020). [(18)F]AZD2461, an Insight on Difference in PARP Binding Profiles for DNA Damage Response PET Imaging. Mol Imaging Biol.

[23] Bowden GD, Stotz S, Kinzler J, Geibel C, Lammerhofer M, Pichler BJ (2021). DoE Optimization Empowers the Automated Preparation of Enantiomerically Pure [(18)F]Talazoparib and its In Vivo Evaluation as a PARP Radiotracer. J Med Chem.

[24] Zhou D, Chen H, Mpoy C, Afrin S, Rogers BE, Garbow JR, et al. Radiosynthesis and evaluation of talazoparib and its derivatives as PARP-1-targeting agents. Biomedicines. 2021;9:565. 10.3390/biomedicines9050565. 10.3390/biomedicines9050565 PMC8157854 34069967

[25] Chan CY, Chen Z, Destro G, Veal M, Lau D, O'Neill E (2022). Imaging PARP with [(18)F]rucaparib in pancreatic cancer models. Eur J Nucl Med Mol Imaging.

[26] Stotz S, Kinzler J, Nies AT, Schwab M, Maurer A (2022). Two experts and a newbie: [(18)F]PARPi vs [(18)F]FTT vs [(18)F]FPyPARP-a comparison of PARP imaging agents. Eur J Nucl Med Mol Imaging.

[27] Ambur Sankaranarayanan R, Kossatz S, Weber W, Beheshti M, Morgenroth A, Mottaghy FM. Advancements in PARP1 targeted nuclear imaging and theranostic probes. J Clin Med. 2020;9:2130. 10.3390/jcm9072130. 10.3390/jcm9072130 PMC7408801 32640708

[28] Puentes LN, Makvandi M, Mach RH (2021). Molecular Imaging: PARP-1 and Beyond. J Nucl Med.

[29] Kaoutzanis C, Chang MC, Abdul Khalek FJ, Kreske E. Non-umbilical cutaneous metastasis of a pancreatic adenocarcinoma. BMJ Case Rep. 2013;2013:bcr2012007931. 10.1136/bcr-2012-007931. 10.1136/bcr-2012-007931 PMC3604162 23307465

[30] Motoshima S, Irie H, Nakazono T, Kamura T, Kudo S (2011). Diffusion-weighted MR imaging in gynecologic cancers. J Gynecol Oncol.

[31] Fujii S, Atsusue E, Kanasaki Y, Kanamori Y, Nakanishi J, Sugihara S (2008). Detection of peritoneal dissemination in gynecological malignancy: evaluation by diffusion-weighted MR imaging. Eur Radiol.

[32] Velikyan I (2014). Prospective of Ga-68-Radiopharmaceutical Development. Theranostics.

[33] Hu KZ, Li JQ, Wang LJ, Huang Y, Li L, Ye SM (2022). Preclinical evaluation and pilot clinical study of F-18 AlF-labeled FAPI-tracer for PET imaging of cancer associated fibroblasts. Acta Pharm Sin B.

[34] Jiang CJ, Tian QW, Xu XP, Li PL, He SM, Chen J, et al. Enhanced antitumor immune responses via a new agent I-131 -labeled dual-target immunosuppressant. Eur J Nucl Med Mol Imaging. 2023;50:275–286. 10.1007/s00259-022-05986-4. 10.1007/s00259-022-05986-4 PMC9816240 36242616

[35] Menear KA, Adcock C, Boulter R, Cockcroft XL, Copsey L, Cranston A (2008). 4–3-(4-Cyclopropanecarbonylpiperazine-1-carbonyl)-4-fluorobenzyl -2H-ph thalazin-1-one: A Novel Bioavailable Inhibitor of Poly(ADP-ribose) Polymerase-1. J Med Chem.

[36] Dawicki-McKenna JM, Langelier MF, DeNizio JE, Riccio AA, Cao CD, Karch KR (2015). PARP-1 Activation Requires Local Unfolding of an Autoinhibitory Domain. Mol Cell.

[37] Salinas B, Irwin CP, Kossatz S, Bolaender A, Chiosis G, Pillarsetty N (2015). Radioiodinated PARP1 tracers for glioblastoma imaging. EJNMMI Res.

[38] Kim G, Ison G, McKee AE, Zhang H, Tang SH, Gwise T (2015). FDA Approval Summary: Olaparib Monotherapy in Patients with Deleterious Germline BRCA-Mutated Advanced Ovarian Cancer Treated with Three or More Lines of Chemotherapy. Clin Cancer Res.

[39] Chen Y, Zhang L, Hao Q (2013). Olaparib: a promising PARP inhibitor in ovarian cancer therapy. Arch Gynecol Obstet.

