# Supplementary material for: PET imaging of PARP expression using 68Ga-labelled inhibitors
Source: Eur J Nucl Med Mol Imaging. 2023 May 5;50(9):2606–20. doi: 10.1007/s00259-023-06249-6 (PMC10317875; doi:10.1007/s00259-023-06249-6)
Supplement: Supplementary file 1 — Supplementary file1 (PDF 7876 KB) [file 259_2023_6249_MOESM1_ESM.pdf]

Supplemental Materials

PET imaging of PARP-1 expression using ^{68}Ga-labeled inhibitors

Authors: Xiangwei Wang[1,2*], Wei Liu[1,2*], Ke Li[1,2*], Kaiwen Chen[3], Simin He[1,2], Jianping Zhang[1,2], Bingxin Gu[1,2], Xiaoping Xu[1,2], and Shaoli Song[1,2]

[1]Department of Nuclear Medicine, Fudan University Shanghai Cancer Center, Shanghai, China 200032

[2]Shanghai Engineering Research Center of Molecular Imaging Probes, Shanghai, China 200032

[3]College of Chemistry and Materials Science, Shanghai Normal University

Corresponding authors:

Shaoli Song, MD

Department of Nuclear Medicine, Fudan University Shanghai Cancer Center, Shanghai 200032, China; Shanghai Engineering Research Center of Molecular Imaging Probes, Shanghai 200032, China

E-mail: shaoli-song@163.com

Xiaoping Xu, PhD

Department of Nuclear Medicine, Fudan University Shanghai Cancer Center, Shanghai 200032, China; Shanghai Engineering Research Center of Molecular Imaging Probes, Shanghai 200032, China

E-mail: xxp0012@ustc.edu

Materials and methods

Materials. Unless otherwise specified, all reactions were carried out under the atmosphere. All common reagents or materials were purchased from commercial sources and used without further purification. Olaparib, DOTA-tris(t-Bu ester) (Compound **2**), Boc-GAMMA-ABU-OH **(**Compound **4),** and Boc-(Gly)$_3$-OH **(**Compound **8)** were obtained from energy-chemical in Shanghai, China. 4-(4-fluoro-3-(piperazine-1-carbonyl)benzyl)phthalazin-1(2H)-one (Compound **1)** was purchased from the commercial source Macklin in Tianjin, China. Flash chromatography was performed using silica gel from Qingdao Haiyang, and its specification was 300-400 mesh.

Equipment. NMR spectra were recorded on Bruker AMX 400 spectrometer, Bruker AMX 500 spectrometer, and calibrated using residual deuterated solvent as an internal reference (DMSO-d_6: 2.49 ppm for ^{1}H NMR). All analytical MS data were recorded on Electron Spray Ionization (ESI).

The ^{68}Ge-^{68}Ga generator was obtained from Eckert & Ziegler (Berlin, Germany). The radiopharmaceuticals were analyzed by High-performance liquid chromatography (HPLC) and radioactive thin layer chromatography (TLC; Raytest mini-GITA). HPLC analyses were performed on an Agilent 1200 system with a flow-through γ-detector (Raytest GABI).

The analytical HPLC conditions are described in methods 1-2.

Compounds DOTA-Olaparib, ^{68}Ga-DOTA-Olaparib, DOTA-GABA-Olaparib, and ^{68}Ga-DOTA-GABA-Olaparib were analyzed by method 1: Kinetex 5 µm particles C_{18} 100A 250 * 4.6 mm (00G-4633-E0), 5-64% acetonitrile (0.1% trifluoroacetic acid (TFA)) in H_2O (0.1% TFA) in a

linear gradient over 12 min, 1 mL/min, UV = 254 nm.

Compounds DOTA-(Gly)3-Olaparib and ^{68}Ga-DOTA-(Gly)3-Olaparib were analyzed by method 2: Kinetex 5 µm particles C_{18} 100A 250 * 4.6 mm (00G-4633-E0), 5-95% acetonitrile (0.1% TFA) in H_2O (0.1% TFA) in a linear gradient over 10 min, 1 mL/min, UV = 254 nm.

The imaging studies were performed on an Inveon PET/CT (Siemens Medical Solutions) and reconstructed using Inveon Research (Siemens Medical Solutions). Radioactivity was measured with a γ-counter (SN-697, Shanghai Nuclear Institute Rihuan Photoelectric Instrument Company Limited).

SK-OV-3 and A549 cells were obtained from the Shanghai Institute of Biochemistry and Cell Biology, Chinese Academy of Sciences. Normal (CD-1(ICR)IGS) mice and BALB/c nude mice aged 6-8 weeks were obtained from Charles River (Zhejiang, China) and Ling Chang Biotechnology Company Limited (Shanghai, China), respectively.

SK-OV-3 and A549 cells were grown as a monolayer using DMEM (Gibco, Carlsbad, USA) and RPMI 1640 medium (Gibco, Carlsbad, USA) containing 10% foetal bovine serum (FBS) (Gibco, Carlsbad, USA) and 1% penicillin–streptomycin (Invitrogen, Carlsbad, USA) at 37 °C in a humidified atmosphere with 5% CO_2 and 95% air.

Chemical synthesis

2.1.1. Synthesis of compound ^{68}Ga-DOTA-Olaparib

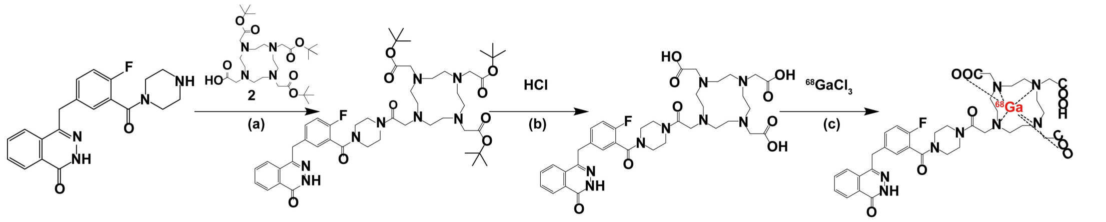

Fig. S1 Synthetic route and speculative structures of ^{68}Ga-DOTA-Olaparib. Reagents and conditions: (a) HATU, DIPEA, DMF, r.t.. (b) 12 N HCl, r.t.. (c) $^{68}GaCl_3$, NaAc, 100 °C, 10 min

Synthesis of compound 3: 2-[4,7,10-tris[2-[(2-methylpropan-2-yl)oxy]-2-oxoethyl]-1,4,7,10-tetrazacyclododec-1-yl]acetic acid (**2**, 304.8 mg, 0.532 mmol, 1.3 eq), HATU (171.23 mg, 0.450 mmol, 1.1 eq) and DIPEA (137.57 mg, 1.06 mmol, 2.6 eq) was dissolved in 7 mL of anhydrous DMF. The reaction mixture was allowed to be stirred at room temperature for 0.5 h. A mixture of 4-(4-fluoro-3-(piperazine-1-carbonyl)benzyl)phthalazin-1(2H)-one (**1**, 150 mg, 0.409 mmol, 1 eq) in 3 mL of anhydrous DMF were added to the solution. The reaction mixture was allowed to be stirred at room temperature overnight. TLC was utilized to monitor the reaction process. The solvent was evaporated under vacuum. The reaction mixture was diluted with ethyl acetate (50 mL), and the whole was extracted with saturated $NaHCO_3$ (2 x 30 mL). The organic phase was dried over anhydrous Na_2SO_4, filtered, and evaporated under vacuum. The crude product was purified by column chromatography to yield the desired product **3** (168 mg, 44.56%) as a solid powder.

^{1}H NMR (500 MHz, DMSO-d_6) δ: 12.62 (s, 1H), 8.27 (s, 1H), 7.96 (d, J = 8.1 Hz, 1H), 7.86 (ddt, J = 23.8, 15.2, 7.2 Hz, 2H), 7.47 (dd, J = 12.5, 5.4 Hz, 1H), 7.37 – 7.18 (m, 2H), 4.34 (s, 2H), 3.30 (s, 2H), 3.19 (s, 6H), 2.43 (t, J = 8.1 Hz, 1H), 2.28 – 1.81 (m, 8H), 1.59 – 1.37 (m, 27H).

MS-ESI(+) calcd for $C_{36}H_{45}FN_8O_9$: 920.5172, $[M+H]^+$ found: 921.5, $[M+Na]^+$ found: 943.5.

Synthesis of compound Olaparib-DOTA: Compound **3** (100 mg, 0.109 mmol) was dissolved in

10 mL of concentrated hydrochloric acid. The reaction mixture was stirred at room temperature for 1.5 h. TLC was utilized to monitor the reaction process. The solvent was evaporated under vacuum, ether was added for washing, and the remaining HCl was removed under ultrasonic suction filtration to yield the desired product **Olaparib-DOTA** (78 mg, 95.44%) as a solid powder.

^{1}H NMR (500 MHz, DMSO-d_6) δ: 12.64 (d, J = 2.9 Hz, 1H), 8.26 (d, J = 7.6 Hz, 1H), 7.97 (d, J = 7.5 Hz, 1H), 7.89 (q, J = 7.3 Hz, 1H), 7.84 (d, J = 7.4 Hz, 1H), 7.46 (s, 1H), 7.36 (d, J = 6.3 Hz, 1H), 7.26 (d, J = 9.2 Hz, 1H), 4.34 (s, 4H), 3.69 (d, J = 25.8 Hz, 6H), 3.63 – 3.41 (m, 12H), 3.26 (d, J = 23.2 Hz, 10H), 3.16 (s, 2H).

MS-ESI(+) calcd for $C_{36}H_{45}FN_8O_9$: 752.3294, $[M+H]^+$ found: 753.3.

2.1.2. Synthesis of compound ^{68}Ga-DOTA-GABA-Olaparib

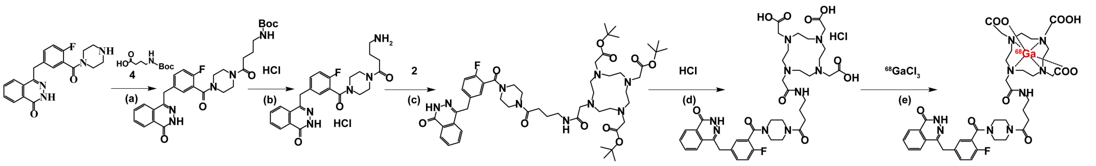

Fig. S2 Synthetic route and speculative structures of ^{68}Ga-DOTA-GABA-Olaparib. Reagents and conditions: (a) HATU, DIPEA, DMF, r.t.. (b) 4 N HCl, r.t.. (c) HATU, DIPEA, DMF, r.t.. (d) 6 N HCl, r.t.. (e) $^{68}GaCl_3$, NaAc, 100 °C, 10 min

Synthesis of compound 5: Boc-GAMMA-ABU-OH (**4**, 72.11 mg, 0.355 mmol, 1.3 eq), HATU (114.15 mg, 0.300 mmol, 1.1 eq), and DIPEA (91.72 mg, 0.710 mmol, 2.6 eq) were dissolved in 7 mL of anhydrous DMF. The reaction mixture was allowed to be stirred at room temperature for 0.5 h. A mixture of 4-(4-fluoro-3-(piperazine-1-carbonyl)benzyl)phthalazin-1(2H)-one (**1**, 100 mg, 0.273 mmol, 1 eq) in 3 mL of anhydrous DMF was added to the solution. The reaction mixture was allowed to be stirred at room temperature overnight. TLC was utilized to monitor the reaction process. The solvent was evaporated under vacuum. The reaction mixture was diluted with ethyl acetate (50 mL), and the mixture was extracted with saturated $NaHCO_3$ (2 x 30 mL). The organic phase was dried over anhydrous Na_2SO_4, filtered, and evaporated under vacuum. The crude product was purified by column chromatography to yield the desired product **5** (126 mg, 83.69%) as a solid powder.

^{1}H NMR (400 MHz, DMSO-d_6) δ: 12.56 (s, 1H), 8.22 (d, J = 7.8 Hz, 1H), 7.96-7.73 (m, 3H), 7.36 (d, J = 31.4 Hz, 2H), 7.20 (t, J = 9.0 Hz, 1H), 6.76 (d, J = 7.3 Hz, 1H), 4.29 (s, 2H), 3.56 (d, J = 22.5 Hz, 2H), 3.47 (s, 2H), 3.12 (d, J = 13.5 Hz, 2H), 2.88 (t, J = 6.3 Hz, 2H), 2.25 (dt, J = 28.1, 7.2 Hz, 2H), 1.56 (s, 2H), 1.32 (d, J = 9.7 Hz, 9H).

MS-ESI(+) calcd for $C_{29}H_{34}FN_5O_5$: 551.2544, $[M+Na]^+$ found: 574.2.

Synthesis of compound 6: Compound **5** (108 mg, 0.205 mmol) was dissolved in 10 mL of hydrogen chloride solution 4.0 M in dioxane. The reaction mixture was stirred at room temperature for 3 h. TLC was utilized to monitor the reaction process. The solvent was evaporated under vacuum, ether was added for washing, and the remaining HCl was removed under ultrasonic suction filtration to yield the desired product **6** (85 mg, 91.60%) as a solid powder.

^{1}H NMR (500 MHz, DMSO-d_6) δ: 12.64 (d, J = 3.8 Hz, 1H), 8.28 – 8.21 (m, 4H), 7.97 (d, J = 8.0 Hz, 1H), 7.89 (d, J = 9.6 Hz, 1H), 7.84 (d, J = 7.6 Hz, 1H), 7.69 (d, J = 27.3 Hz, 1H), 7.45 (t, J = 6.8 Hz, 1H), 7.38 (d, J = 6.6 Hz, 1H), 7.23 (t, J = 8.5 Hz, 1H), 4.33 (s, 2H), 3.88 (s, 2H), 3.66 (d, J =

5.2 Hz, 1H), 3.58 (s, 1H), 3.53 (d, J = 5.0 Hz, 2H), 3.40 – 3.37 (m, 2H), 3.26 – 3.11 (m, 2H), 2.80 (t, J = 6.5 Hz, 2H), 2.47 (d, J = 6.8 Hz, 1H), 1.83 (d, J = 7.0 Hz, 2H).

MS-ESI(+) calcd for $C_{24}H_{26}FN_5O_3$: 451.2020, $[M+H]^+$ found: 452.2.

Synthesis of compound 7: 2-[4,7,10-tris[2-[(2-methylpropan-2-yl)oxy]-2-oxoethyl]-1,4,7,10-tetrazacyclododec-1-yl]acetic acid (**2**, 131.93 mg, 0.230 mmol, 1.3 eq), HATU (74.11 mg, 0.195 mmol, 1.1 eq), and DIPEA (46.62 mg, 0.461 mmol, 2.6 eq) was dissolved in 7 mL of anhydrous DMF. The reaction mixture was allowed to be stirred at room temperature for 0.5 h. A mixture of compound **6** (80 mg, 0.177 mmol, 1 eq) in 3 mL of anhydrous DMF was added to the solution. The reaction mixture was allowed to be stirred at room temperature overnight. TLC was utilized to monitor the reaction process. The solvent was evaporated under vacuum. The reaction mixture was diluted with ethyl acetate (50 mL), and the whole was extracted with saturated $NaHCO_3$ (2 x 30 mL). The organic phase was dried over anhydrous Na_2SO_4, filtered, and evaporated under vacuum. The crude product was purified by column chromatography to yield the desired product **7** (102 mg, 60.90%) as a solid powder.

^{1}H NMR (400 MHz, DMSO-d_6) δ: 12.56 (s, 1H), 8.22 (s, 1H), 8.08 (d, J = 5.9 Hz, 1H), 7.91 (s, 1H), 7.88-7.77 (m, 1H), 7.43 (s, 1H), 7.33 (s, 1H), 7.20 (s, 1H), 4.31 (d, J = 11.9 Hz, 2H), 3.72-3.36 (m, 12H), 3.21-2.70 (m, 10H), 2.25 (d, J = 27.6 Hz, 10H), 1.61 (s, 2H), 1.38 (t, J = 8.0 Hz, 29H).

MS-ESI(+) calcd for $C_{52}H_{76}FN_9O_{10}$: 1005.5699, $[M+H]^+$ found: 1006.5.

Synthesis of compound DOTA-GABA-Olaparib: Compound **7** (100 mg, 0.109 mmol) was dissolved in 10 mL of hydrogen chloride solution 6.0 M in ultra-pure water. The reaction mixture was stirred at room temperature for 3 h. TLC was utilized to monitor the reaction process. The solvent was evaporated under vacuum, ether was added for washing, and the remaining HCl was removed under ultrasonic suction filtration to yield the desired product **DOTA-GABA-Olaparib** (55 mg, 65.13%) as a solid powder.

MS-ESI(+) calcd for $C_{40}H_{53}ClFN_9O_{10}$: 873.3558, $[M+H]^+$ found: 874.4.

4.1.3. Synthesis of compound ^{68}Ga-DOTA-(Gly)$_3$-Olaparib

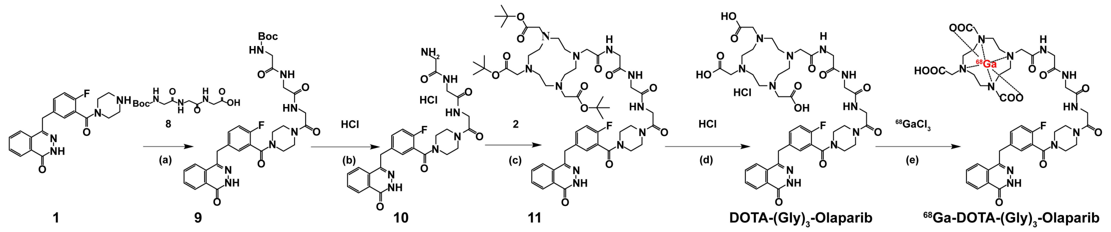

Fig. S3 Synthetic route and speculative structures of ^{68}Ga-DOTA-(Gly)$_3$-Olaparib. Reagents and conditions: (a) HATU, DIPEA, DMF, r.t.. (b) 4 N HCl, r.t.. (c) HATU, DIPEA, DMF, r.t.. (d) 6 N HCl, r.t.. (e) $^{68}GaCl_3$, NaAc, 100 °C, 10 min

Synthesis of compound 9: Boc-(Gly)$_3$-OH (**5**, 102.64 mg, 0.355 mmol, 1.3 eq), HATU (114.15 mg, 0.300 mmol, 1.1 eq), and DIPEA (91.72 mg, 0.710 mmol, 2.6 eq) was dissolved in 7 mL of anhydrous DMF. The reaction mixture was allowed to be stirred at room temperature for 0.5 h. A mixture of 4-(4-fluoro-3-(piperazine-1-carbonyl)benzyl)phthalazin-1(2H)-one (**1**, 100 mg, 0.273 mmol, 1 eq) in 3 mL of anhydrous DMF was added to the solution. The reaction mixture was allowed to be stirred at room temperature overnight. TLC was utilized to monitor the reaction process. The solvent

was evaporated under vacuum. The reaction mixture was diluted with ethyl acetate (50 mL), and the whole was extracted with saturated $NaHCO_3$ (2 x 30 mL). The organic phase was dried over anhydrous Na_2SO_4, filtered, and evaporated under vacuum. The crude product was purified by column chromatography to yield the desired product **9** (108 mg, 62.05%) as a solid powder.

^{1}H NMR (400 MHz, DMSO-d_6) δ: 12.55 (s, 1H), 8.26-8.19 (m, 1H), 8.01 (t, J = 5.8 Hz, 1H), 7.91 (s, 2H), 7.79 (t, J = 7.4 Hz, 2H), 7.40 (td, J = 5.4, 2.6 Hz, 1H), 7.33 (s, 1H), 7.20 (t, J = 9.0 Hz, 1H), 6.95 (t, J = 5.8 Hz, 1H), 4.29 (s, 2H), 4.24 (s, 2H), 3.71 (d, J = 5.7 Hz, 2H), 3.59-3.42 (m, 6H), 3.15 (d, J = 19.6 Hz, 2H), 2.60-2.50 (m, 2H), 1.34 (s, 9H).

MS-ESI(+) calcd for $C_{31}H_{36}FN_7O_7$: 637.2660, $[M+Na]^+$ found: 660.3.

Synthesis of compound 10: Compound **9** (60 mg, 0.205 mmol) was dissolved in 10 mL of hydrogen chloride solution 4.0 M in dioxane. The reaction mixture was stirred at room temperature for 3 h. TLC was utilized to monitor the reaction process. The solvent was evaporated under vacuum, ether was added for washing, and the remaining HCl was removed under ultrasonic suction filtration to yield the desired product **10** (46 mg, 85.17%) as a solid powder.

^{1}H NMR (500 MHz, DMSO-d_6) δ: 12.62 (s, 1H), 8.26 (s, 1H), 8.17 (d, J = 8.0 Hz, 3H), 7.98 (d, J = 8.1 Hz, 1H), 7.91 (q, J = 7.1 Hz, 1H), 7.84 (t, J = 7.6 Hz, 1H), 7.46 (t, J = 7.2 Hz, 1H), 7.38 (s, 1H), 7.25 (t, J = 9.1 Hz, 1H), 4.34 (s, 2H), 4.05 – 3.92 (m, 2H), 3.86 (s, 2H), 3.66 (s, 1H), 3.60 (s, 3H), 3.54 – 3.51 (m, 2H), 3.38 (s, 2H), 3.20 (d, J = 26.0 Hz, 2H).

MS-ESI(-) calcd for $C_{26}H_{28}FN_7O_5$: 537.2136, $[M-H]^-$ found: 536.3， $[M+HCl-H]^-$ found: 572.1.

Synthesis of compound 11: 2-[4,7,10-tris[2-[(2-methylpropan-2-yl)oxy]-2-oxoethyl]-1,4,7,10-tetrazacyclododec-1-yl]acetic acid (**2**, 55.40 mg, 0.097 mmol, 1.3 eq), HATU (31.12 mg, 0.082 mmol, 1.1 eq), and DIPEA (19.58 mg, 0.193 mmol, 2.6 eq) were dissolved in 7 mL of anhydrous DMF. The reaction mixture was allowed to be stirred at room temperature for 0.5 h. A mixture of compound **10** (40 mg, 0.074 mmol, 1 eq) in 3 mL of anhydrous DMF was added to the solution. The reaction mixture was allowed to be stirred at room temperature overnight. TLC was utilized to monitor the reaction process. The solvent was evaporated under vacuum. The reaction mixture was diluted with ethyl acetate (50 mL), and the whole was extracted with saturated $NaHCO_3$ (2 x 30 mL). The organic phase was dried over anhydrous Na_2SO_4, filtered, and evaporated under vacuum. The crude product was purified by column chromatography to yield the desired product **11** (41 mg, 53.86%) as a solid powder.

MS-ESI(+) calcd for $C_{54}H_{78}FN_{11}O_{12}$: 1091.5815, $[M+H]^+$ found: 1092.6, $[M+Na]^+$ found: 1114.5.

Synthesis of compound DOTA-(Gly)$_3$-Olaparib: Compound **11** (40 mg, 0.109 mmol) was dissolved in 10 mL of concentrated hydrochloric acid. The reaction mixture was stirred at room temperature for 1.5 h. TLC was utilized to monitor the reaction process. The solvent was evaporated under vacuum, ether was added for washing, and the remaining HCl was removed under ultrasonic suction filtration to yield the desired product **DOTA-(Gly)$_3$-Olaparib** (28 mg, 82.74%) as a solid powder.

MS-ESI(-) calcd for $C_{42}H_{54}FN_{11}O_{12}$: 923.3937, $[M-H]^-$ found: 922.5, $[M+HCl+H]^+$ found: 960.3.

2.1.4. Synthesis of fluorescence compound FL-Olaparib

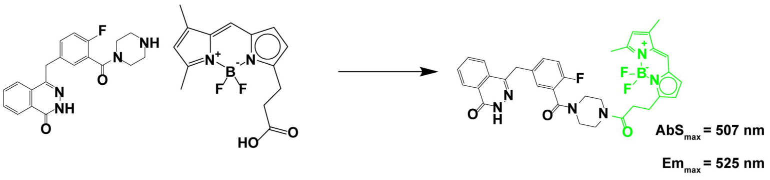

1 3-bodipy-propanoic acid FL-Olaparib

Fig. S4 Synthetic route and speculative structures of FL-Olaparib. Reagents and conditions: HATU, DIPEA, DMF, r.t.

Synthesis of compound FL-Olaparib: 3-bodipy-propanoic acid (38.27 mg, 0.131 mmol, 1.2 eq), HATU (45.66 mg, 0.120 mmol, 1.1 eq), and DIPEA (36.69 mg, 0.284 mmol, 2.6 eq) was dissolved in 4 mL of anhydrous DMF. The reaction mixture was allowed to be stirred at room temperature for 0.5 h. A mixture of 4-(4-fluoro-3-(piperazine-1-carbonyl)benzyl)phthalazin-1(2H)-one (**1**, 40.00 mg, 0.109 mmol, 1 eq) in 3 mL of anhydrous DMF were added to the solution. The reaction mixture was allowed to be stirred at room temperature overnight. TLC was utilized to monitor the reaction process. The solvent was evaporated under vacuum. The reaction mixture was diluted with ethyl acetate (50 mL), and the whole was extracted with saturated $NaHCO_3$ (2 x 30 mL). The organic phase was dried over anhydrous Na_2SO_4, filtered, and evaporated under vacuum. The crude product was purified by column chromatography to yield the desired product **FL-Olaparib** (39 mg, 55.78%) as a solid powder.

^{1}H NMR (400 MHz, DMSO-d_6) δ: 12.56 (s, 1H), 8.22 (d, J = 7.8 Hz, 1H), 7.91 (s, 1H), 7.88-7.71 (m, 1H), 7.44 (dd, J = 17.1, 10.9 Hz, 3H), 7.31 (d, J = 6.4 Hz, 1H), 7.28-7.13 (m, 3H), 5.71 (s, 1H), 4.29 (s, 2H), 3.63 (s, 4H), 3.20 (s, 4H), 2.85 (s, 3H), 2.78-2.59 (m, 3H).

Results

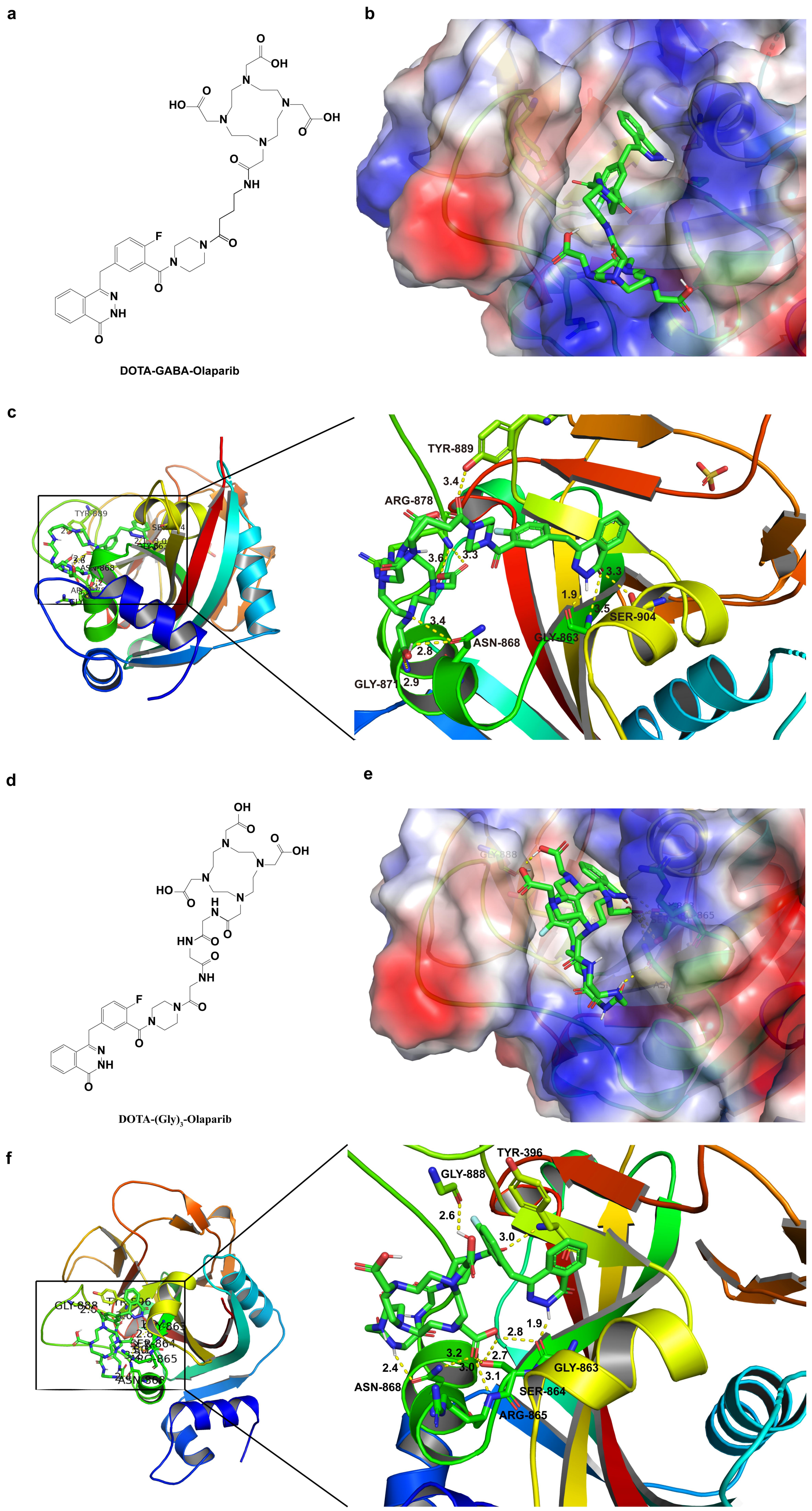

Fig. S5 Design and Binding Mode Analysis of DOTA-GABA-Olaparib and DOTA-$(Gly)_3$-Olaparib

with PARP-1. (a) Chemical structure of DOTA-GABA-Olaparib. (b) Molecular docking structure of DOTA-GABA-Olaparib into PARP-1. (c) Dotted yellow lines labeled H-bonding interactions between DOTA-GABA-Olaparib and PARP-1 residues. (d) Chemical structure of DOTA-(Gly)3-Olaparib. (e) Molecular docking structure of DOTA-(Gly)3-Olaparib into PARP-1. (f) Dotted yellow lines labeled H-bonding interactions between compound DOTA-(Gly)3-Olaparib and PARP-1 residues

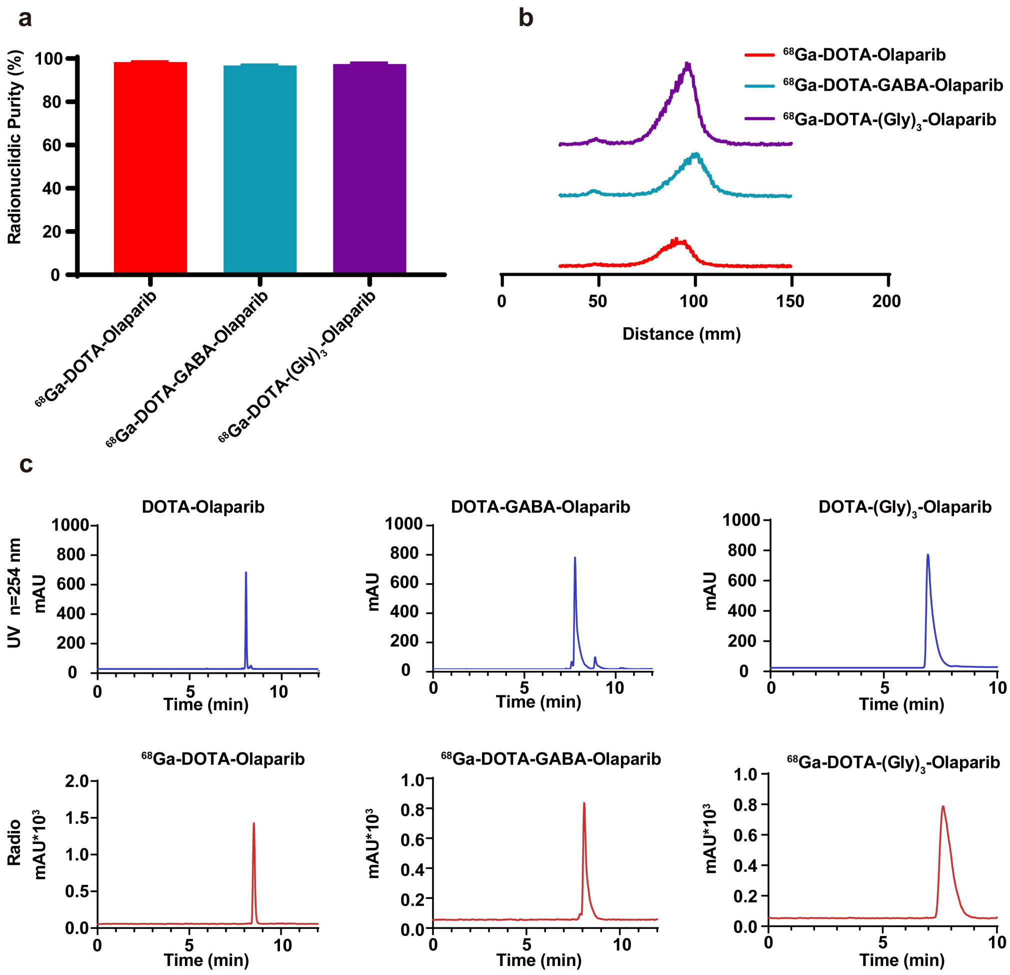

Fig. S6 Characterization of ^{68}Ga-labeled olaparib derivatives. (a) RCP (radiochemical purity) of the three ^{68}Ga-labeled radiotracers using the iTLC-SG(b) A representative iTLC spectrum of the three ^{68}Ga-labeled radiotracers. (c) Representative HPLC spectrum of the three precursors (DOTA-Olaparib, DOTA-GABA-Olaparib, and DOTA-(Gly)3-Olaparib) and the three ^{68}Ga-labeled radiotracers

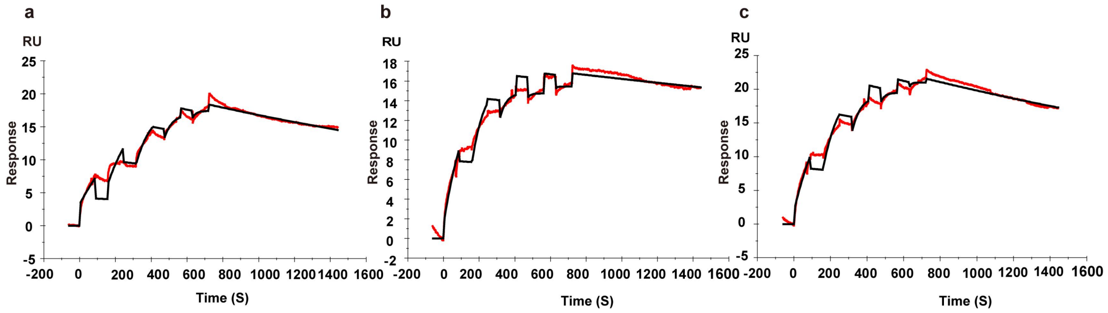

Fig. S7 The binding affinity of DOTA-Olaparib (a), DOTA-GABA-Olaparib (b), and

DOTA-(Gly)3-Olaparib (c) for PARP-1.

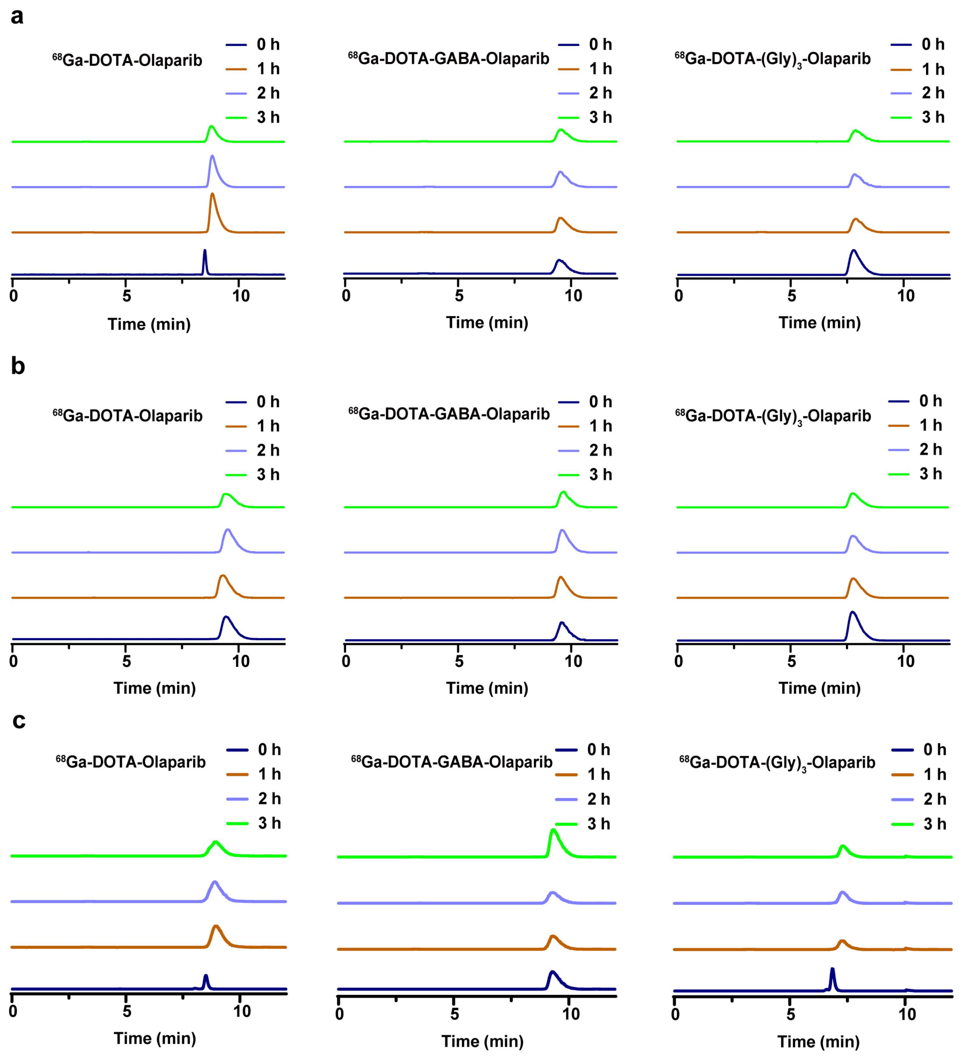

Fig. S8 Stability of the three ^{68}Ga-labeled radiotracers incubated in (a) saline at room temperature for 1, 2, and 3 h and (b) mouse serum *in vitro* at room temperature for 1, 2, and 3 h and (c) human serum *in vitro* at room temperature for 1, 2, and 3 h.

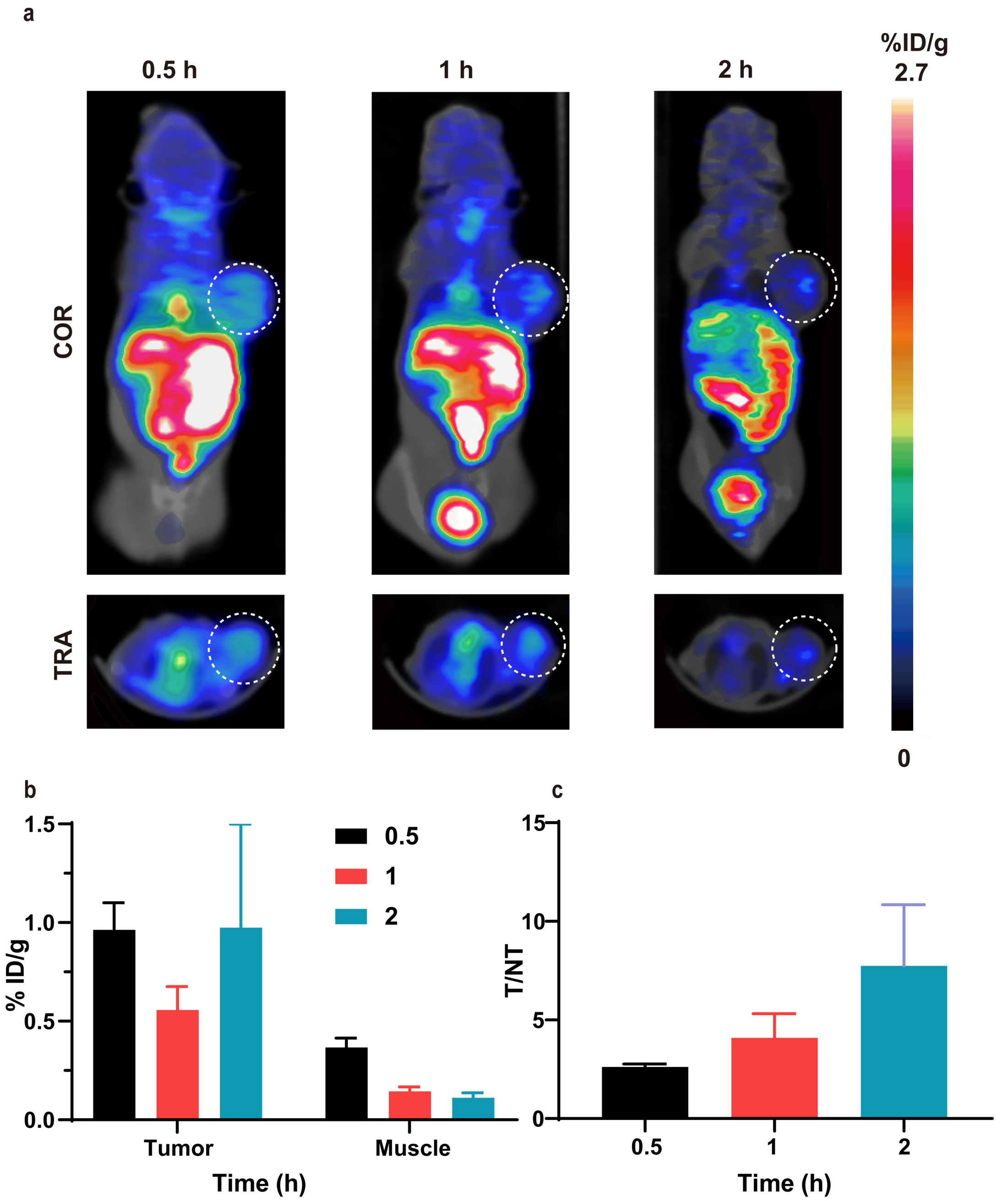

Fig. S9 PET imaging of ^{68}Ga-DOTA-GABA-Olaparib. (a) MicroPET/CT images of ^{68}Ga-DOTA-GABA-Olaparib in SK-OV-3 models at 0.5, 1, and 2 h after radiotracer administration. (b) Quantifying regions of interest (ROI) in SK-OV-3 models expressed as %ID/g. (c) Tumor-to-muscle ratio (n = 3)

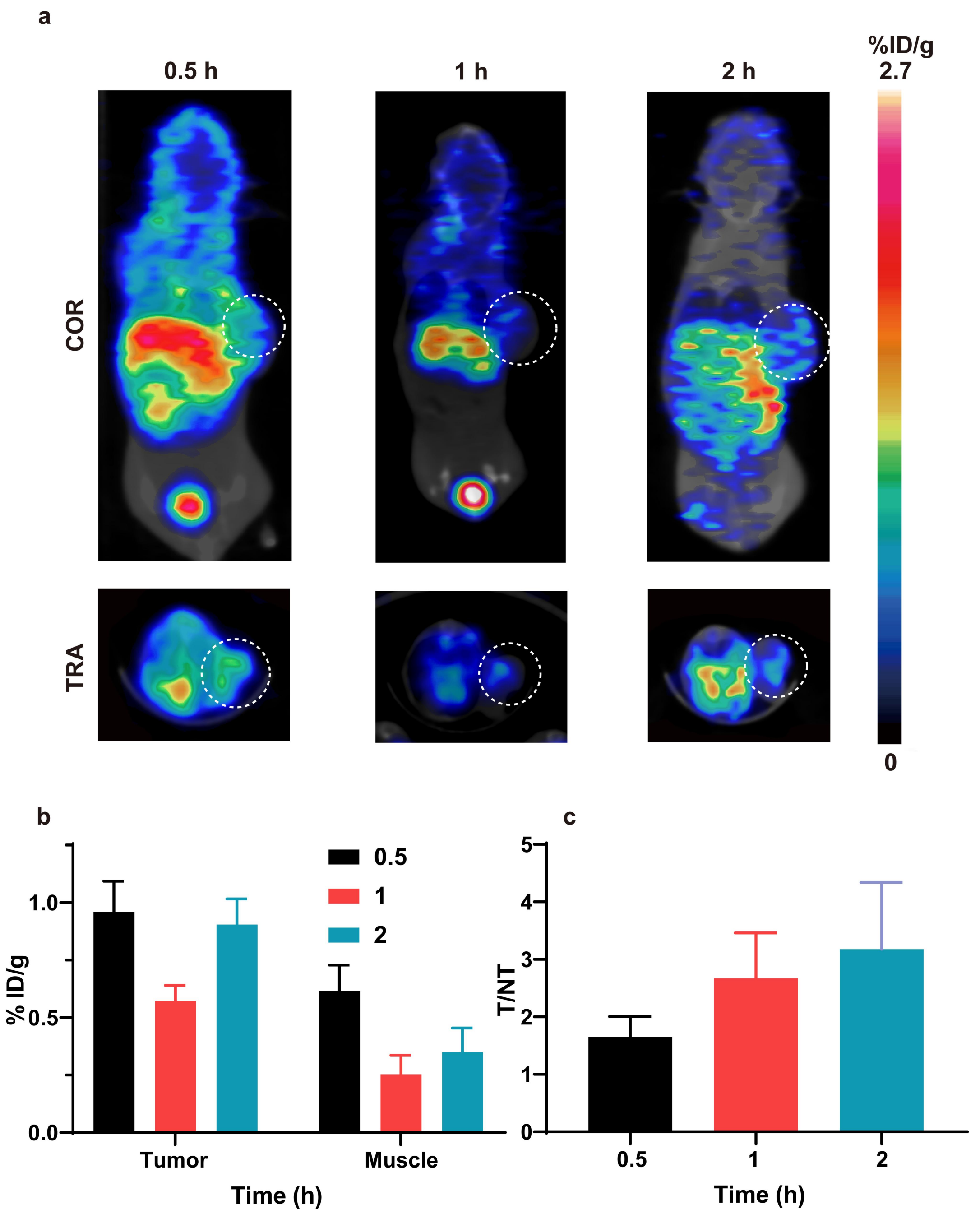

Fig. S10 PET imaging of ^{68}Ga-DOTA-(Gly)$_3$-Olaparib. (a) MicroPET/CT images of ^{68}Ga-DOTA-(Gly)$_3$-Olaparib in SK-OV-3 models at 0.5, 1, and 2 h after radiotracer administration. (b) Quantifying regions of interest (ROI) in SK-OV-3 models expressed as %ID/g. (c) Tumor-to-muscle ratio (n = 3)

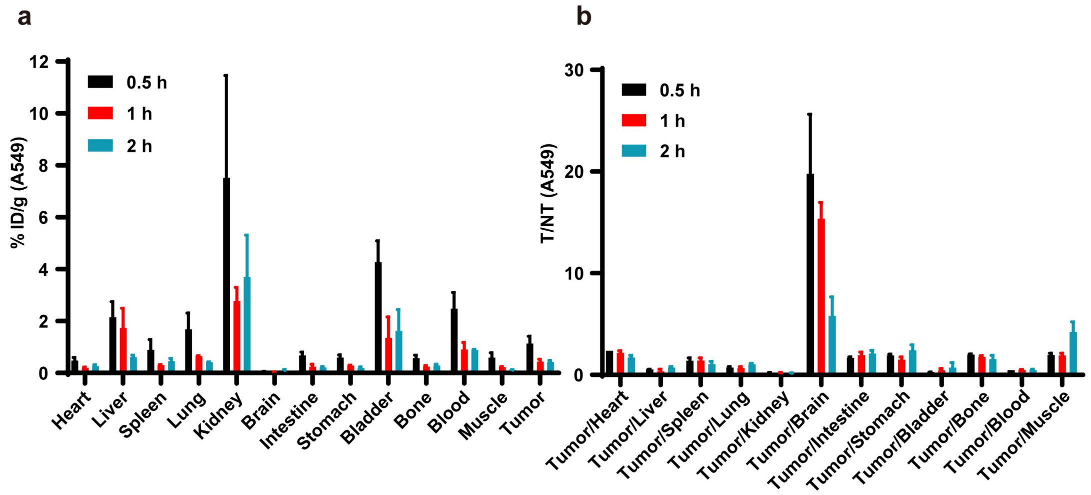

Fig. S11 In vivo efficacy and specificity of ^{68}Ga-DOTA-Olaparib. (a) Biodistribution of ^{68}Ga-DOTA-Olaparib in A549 models (n = 3). (b) The ratios of tumour-to-nontarget tissue (n = 3).

NMR and MS spectra for compounds

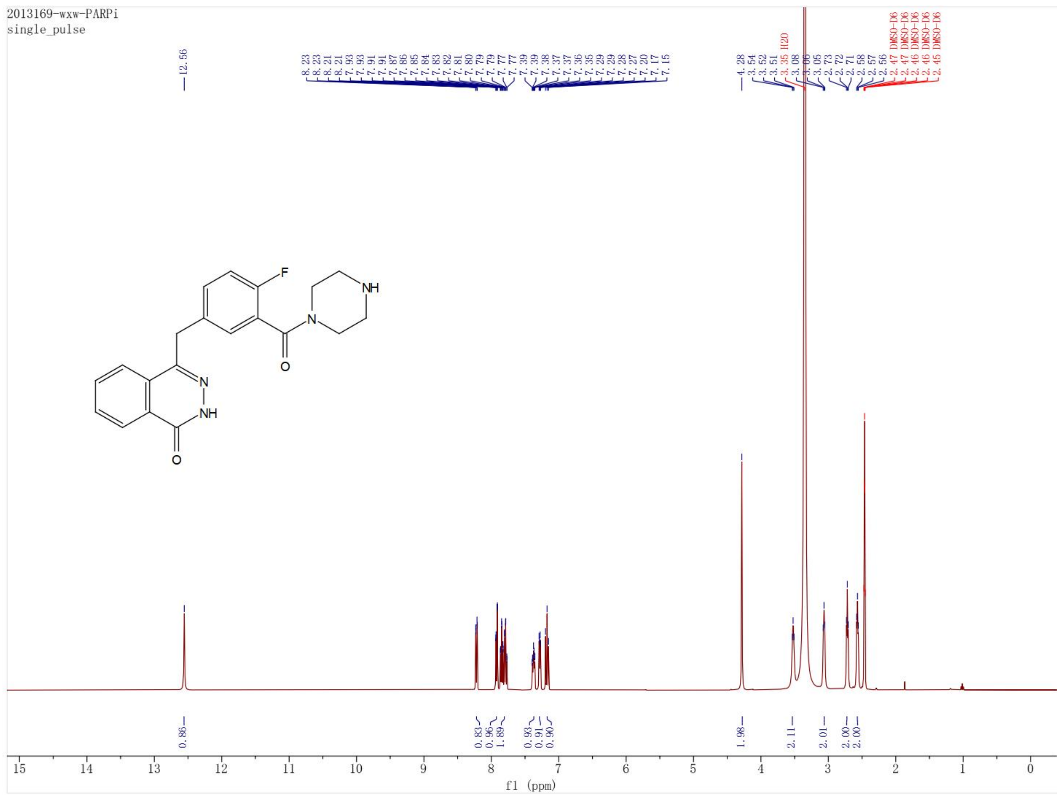

Fig. S12 ^{1}H NMR of compound **1**

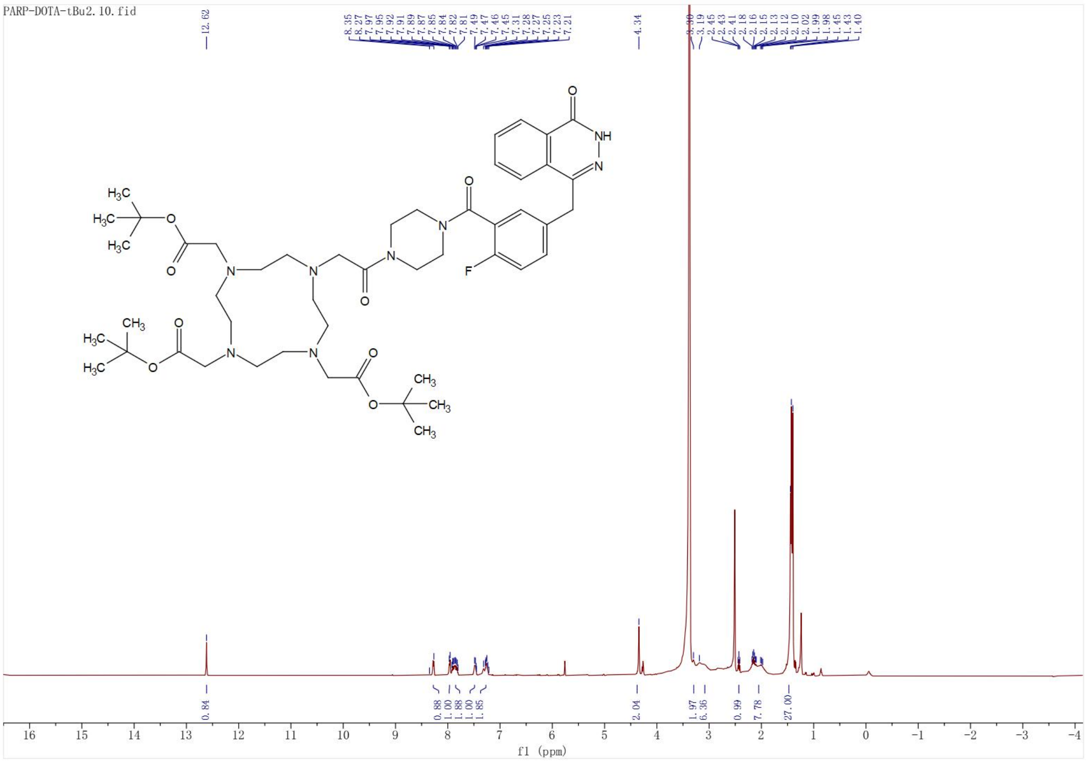

Fig. S13 ^{1}H NMR of compound **3**

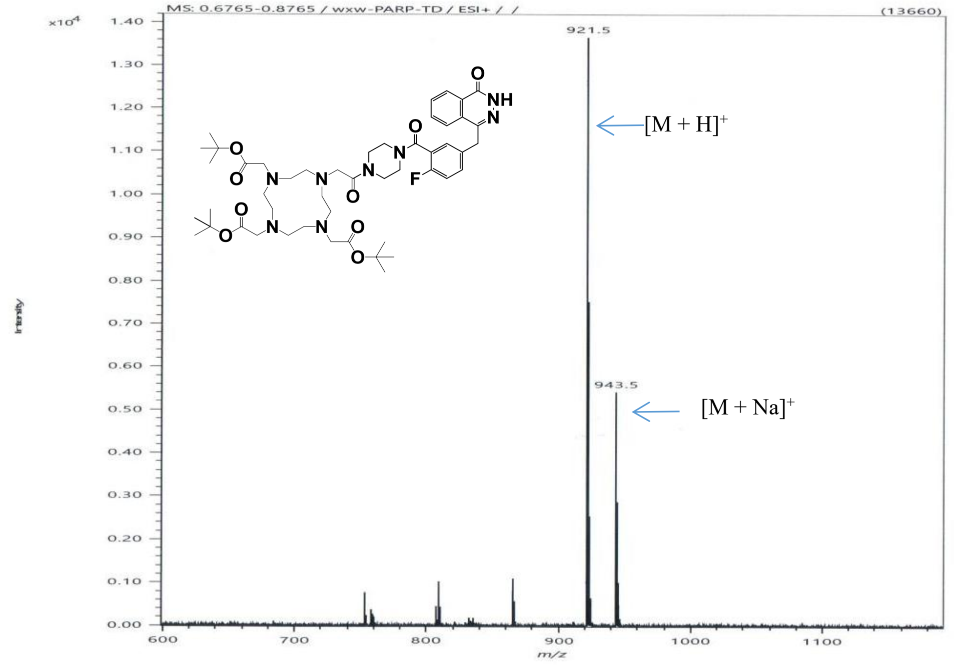

Fig. S14 MS spectrum of compound **3**

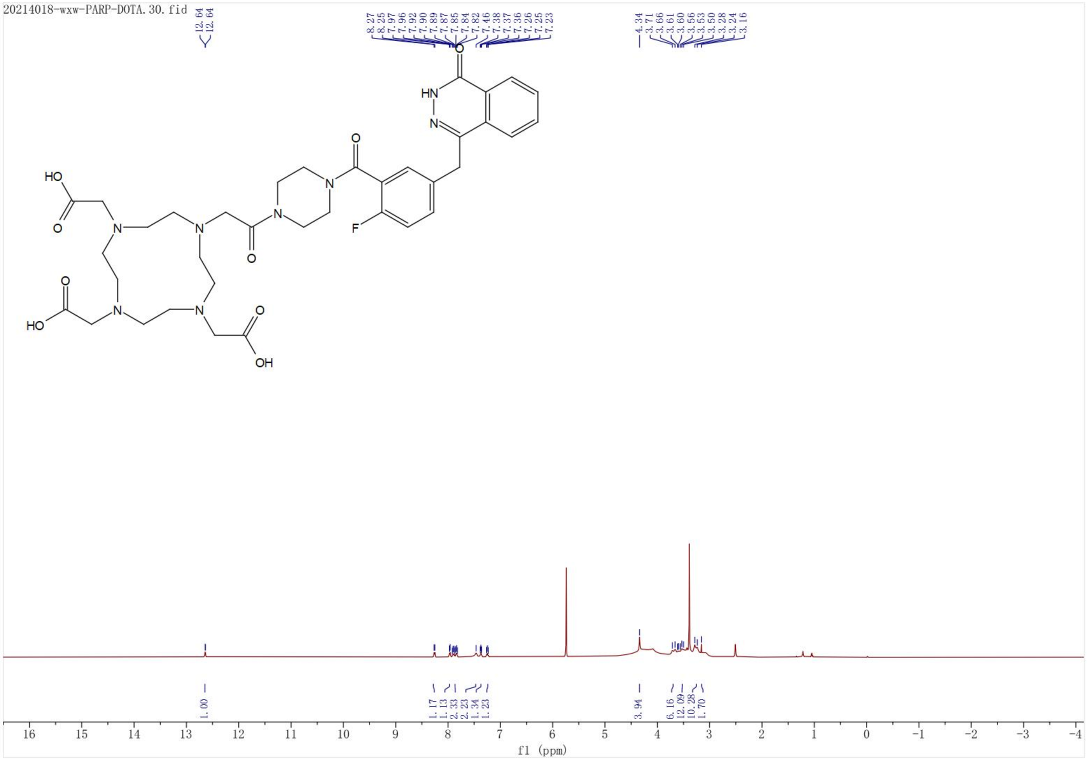

Fig. S15 ^{1}H NMR of compound **DOTA-Olaparib**

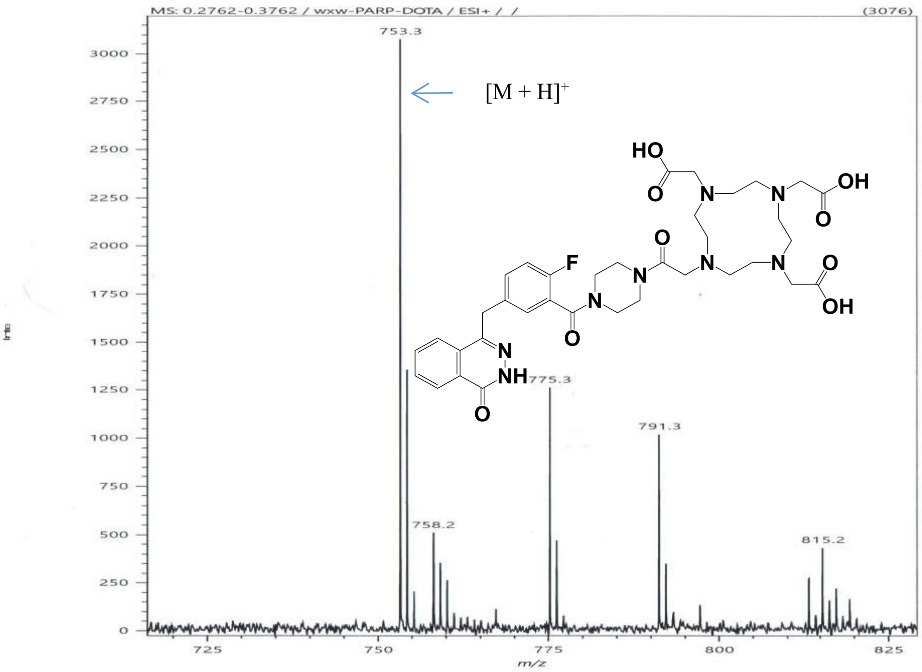

Fig. S16 MS spectrum of compound **DOTA-Olaparib**

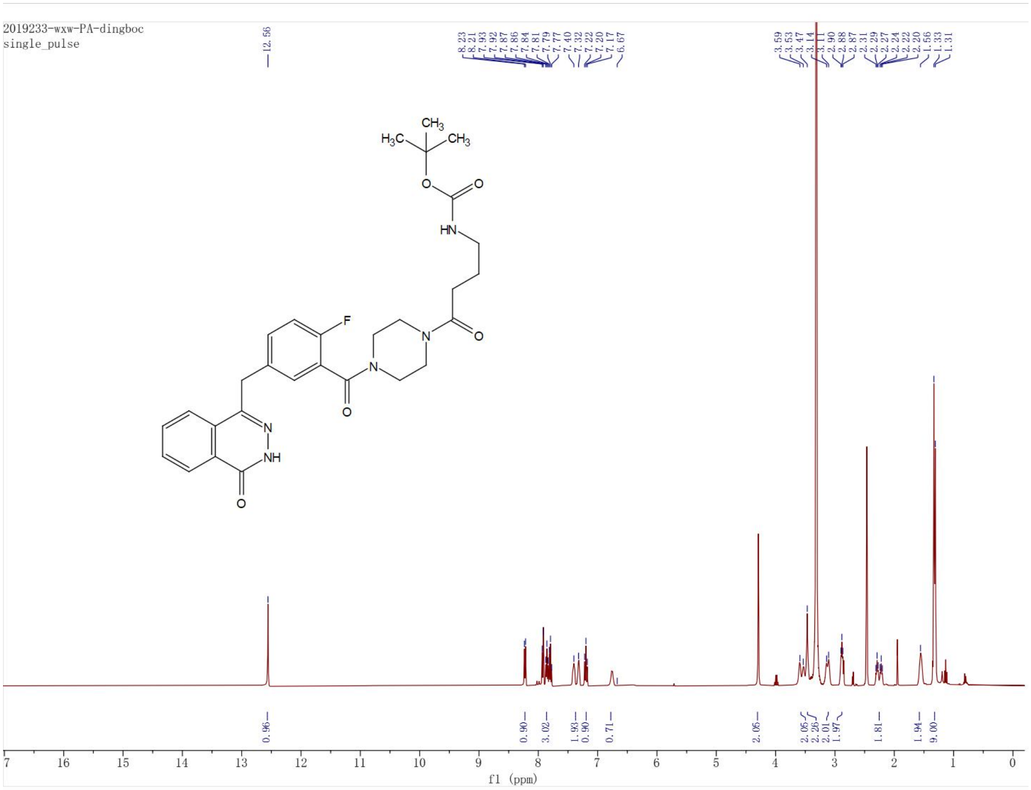

Fig. S17 ^{1}H NMR of compound **5**

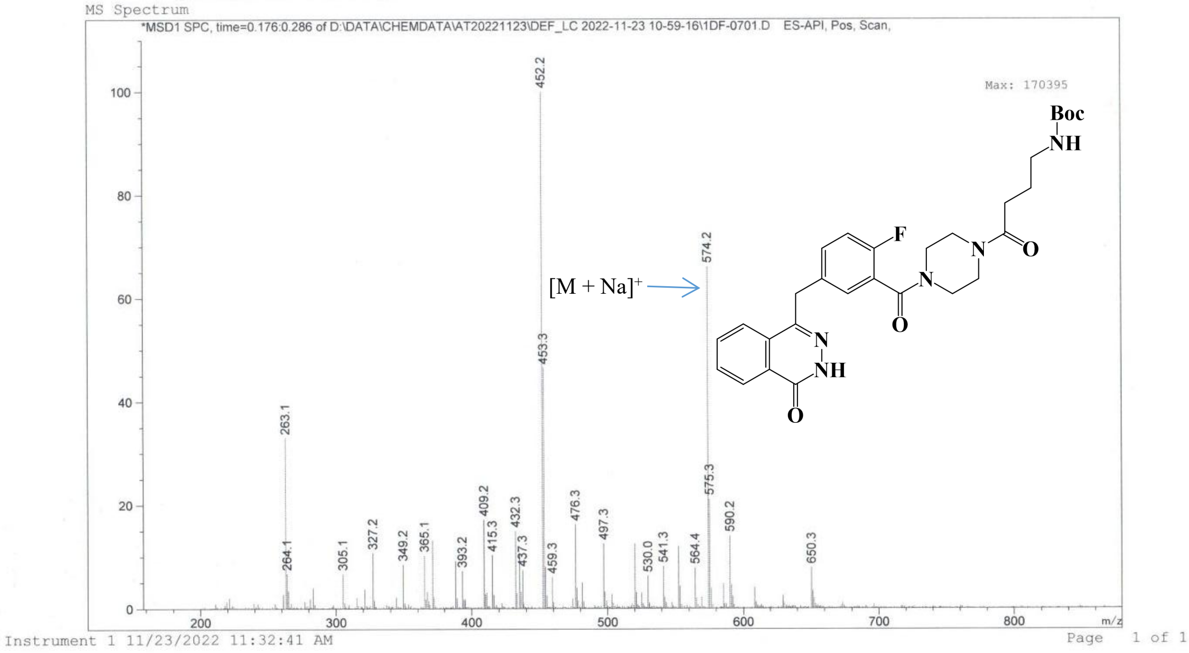

Fig. S18 MS spectrum of compound **5**

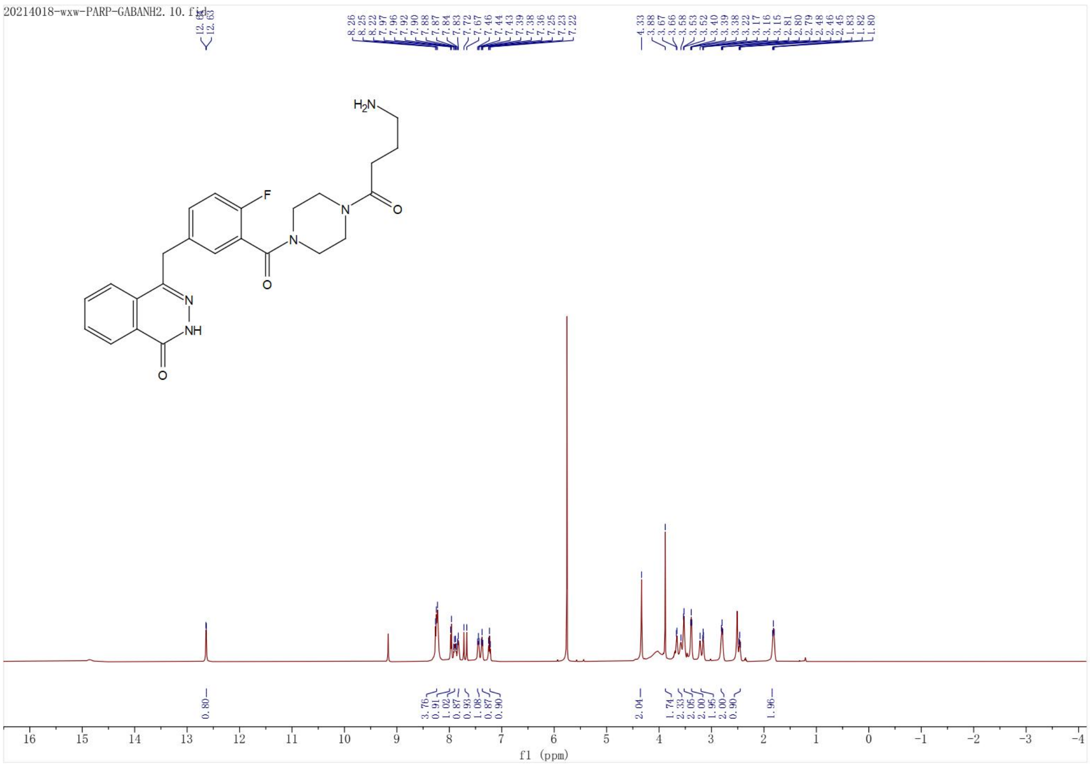

Fig. S19 ^{1}H NMR of compound **6**

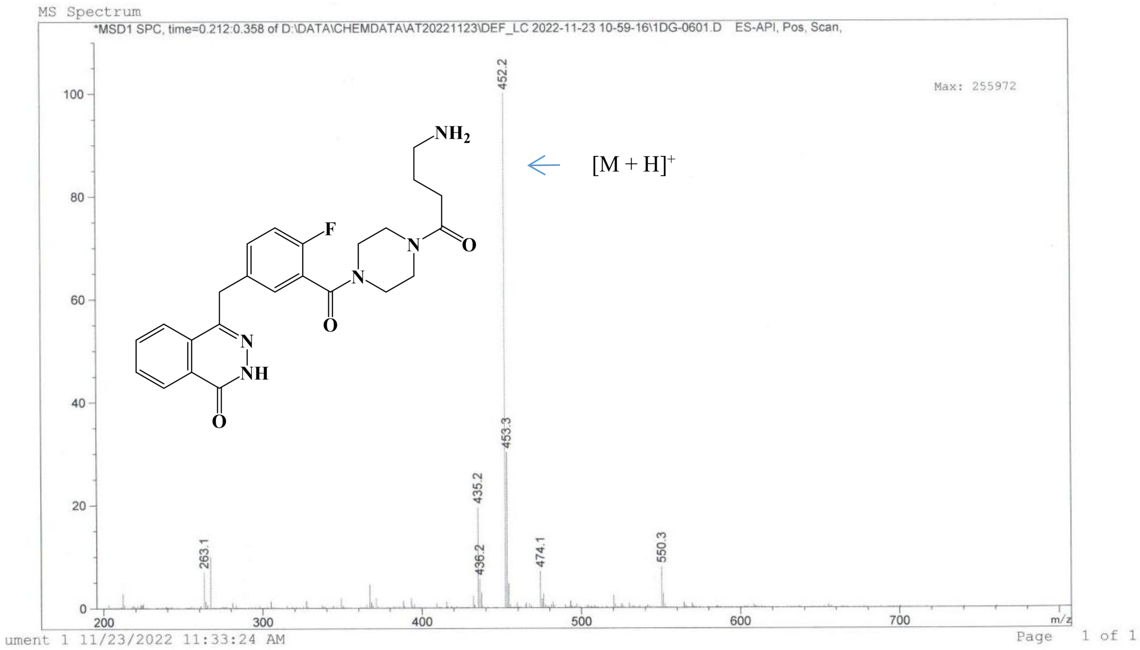

Fig. S20 MS spectrum of compound **6**

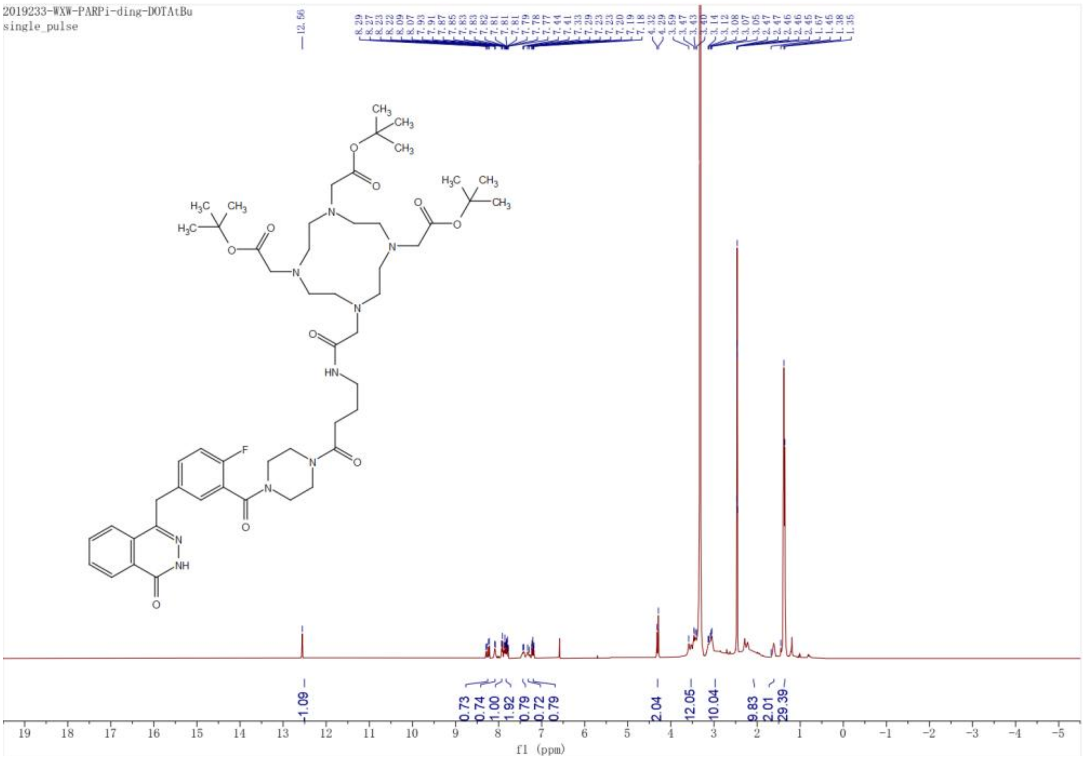

Fig. S21 ^{1}H NMR of compound **7**

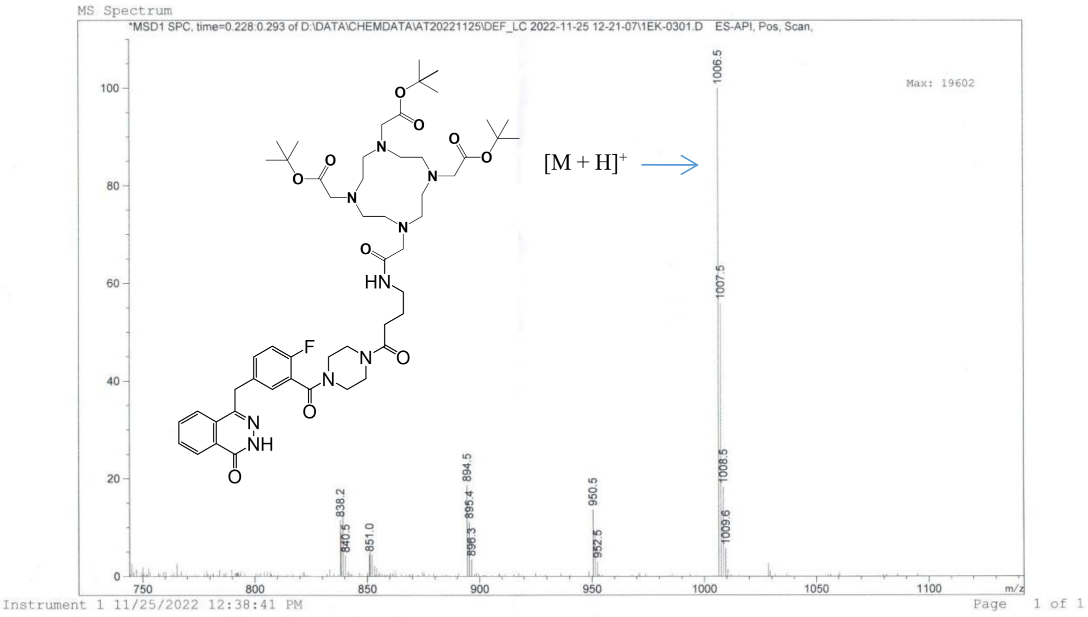

Fig. S22 MS spectrum of compound **7**

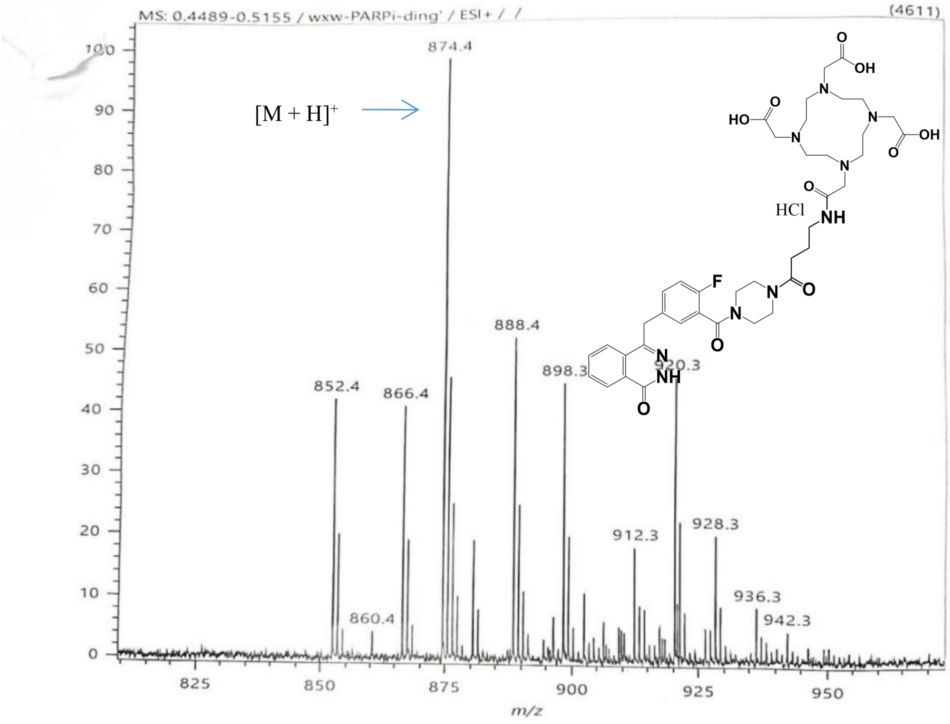

Fig. S23 MS spectrum of compound **DOTA-GABA-Olaparib**

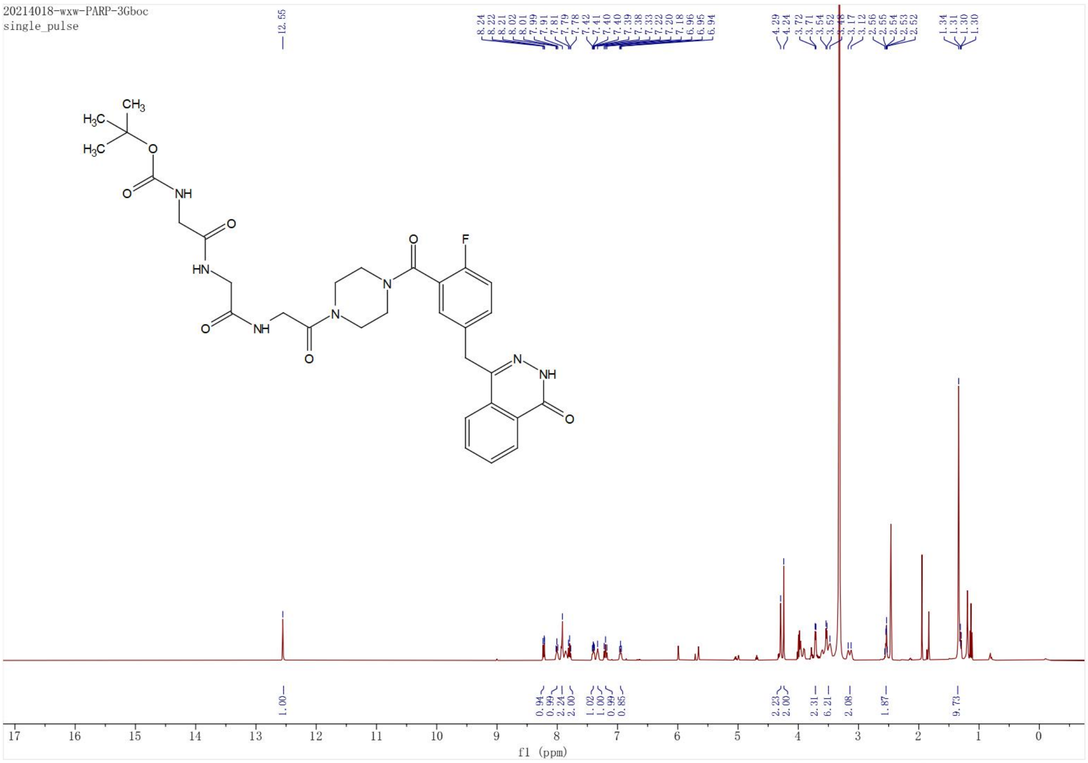

Fig. S24 ^{1}H NMR of compound **9**

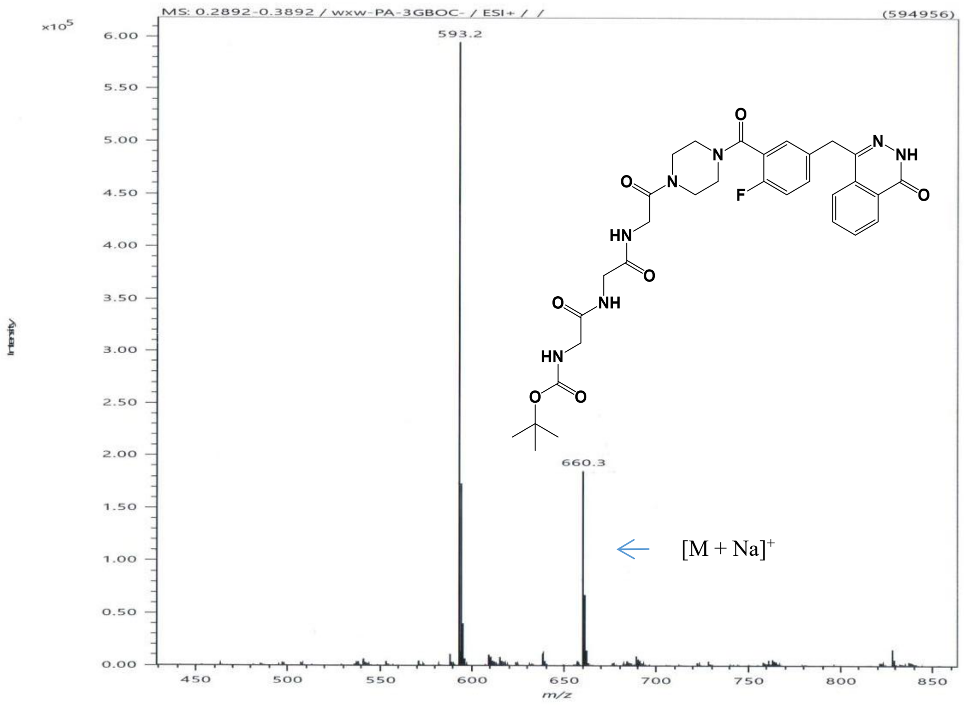

Fig. S25 MS spectrum of compound **9**

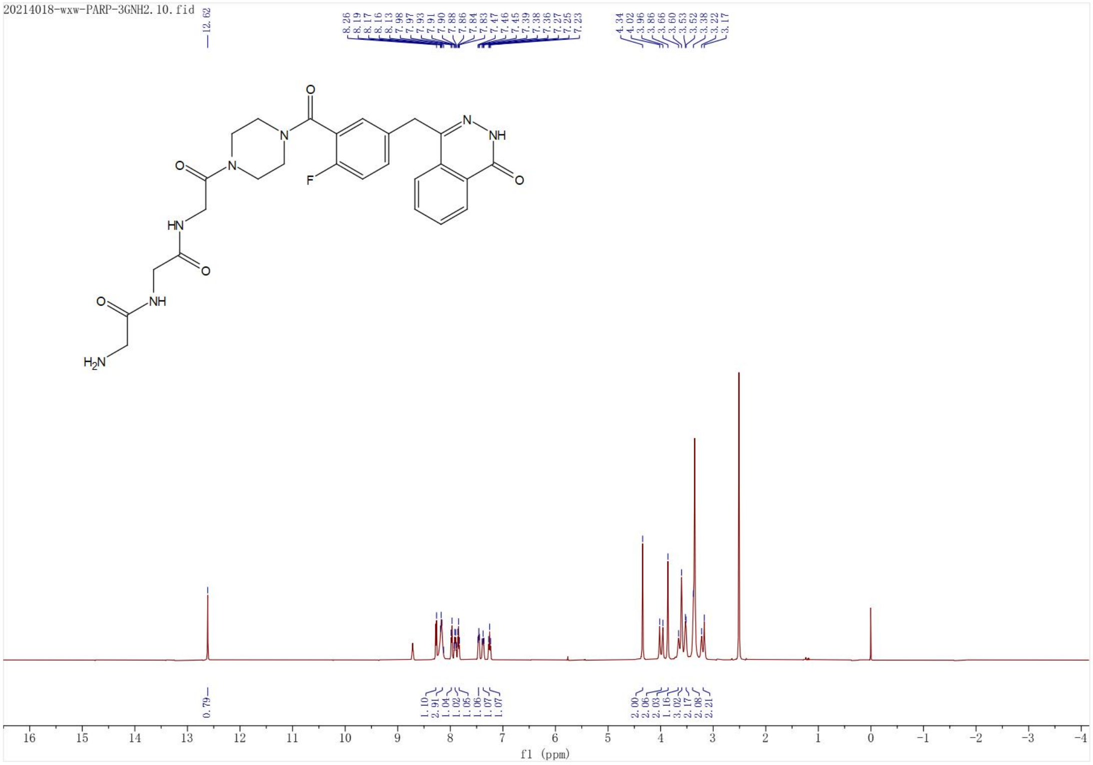

Fig. S26 ^{1}H NMR of compound **10**

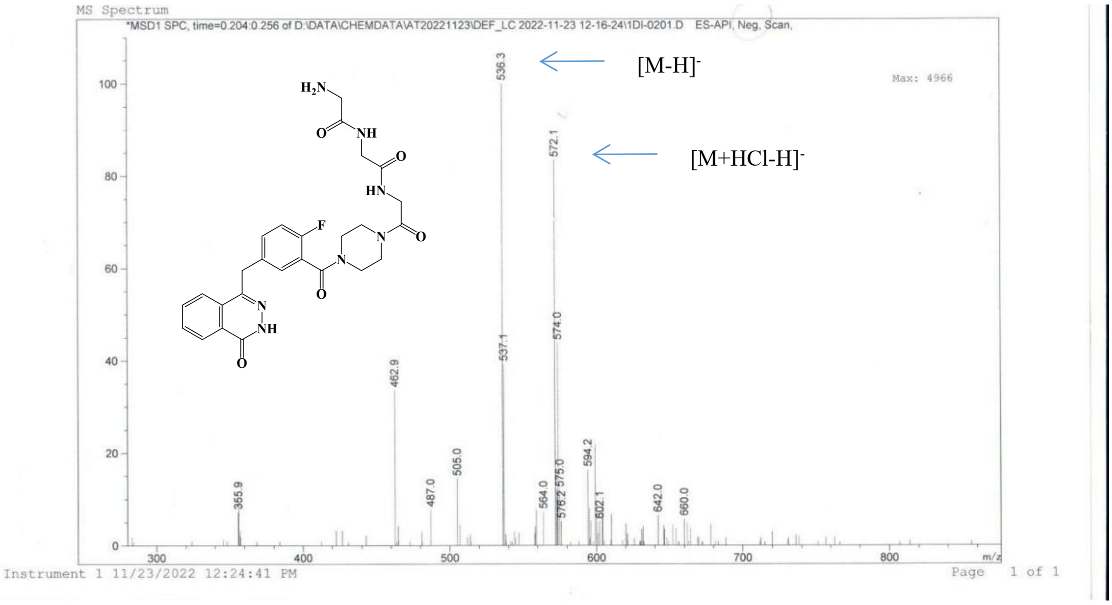

Fig. S27 MS spectrum of compound **10**

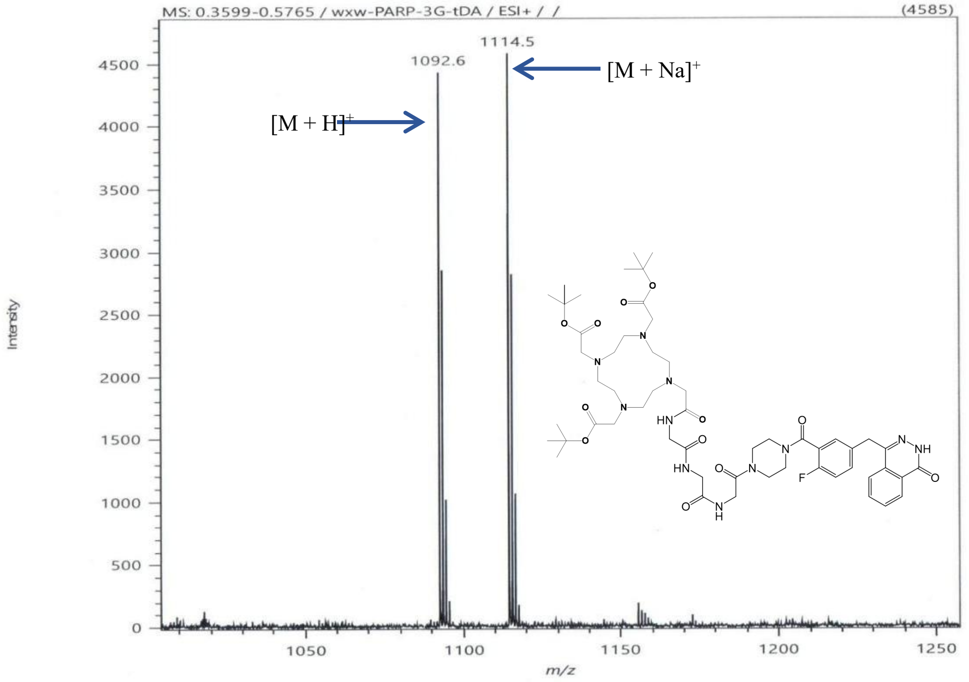

Fig. S28 MS spectrum of compound **11**

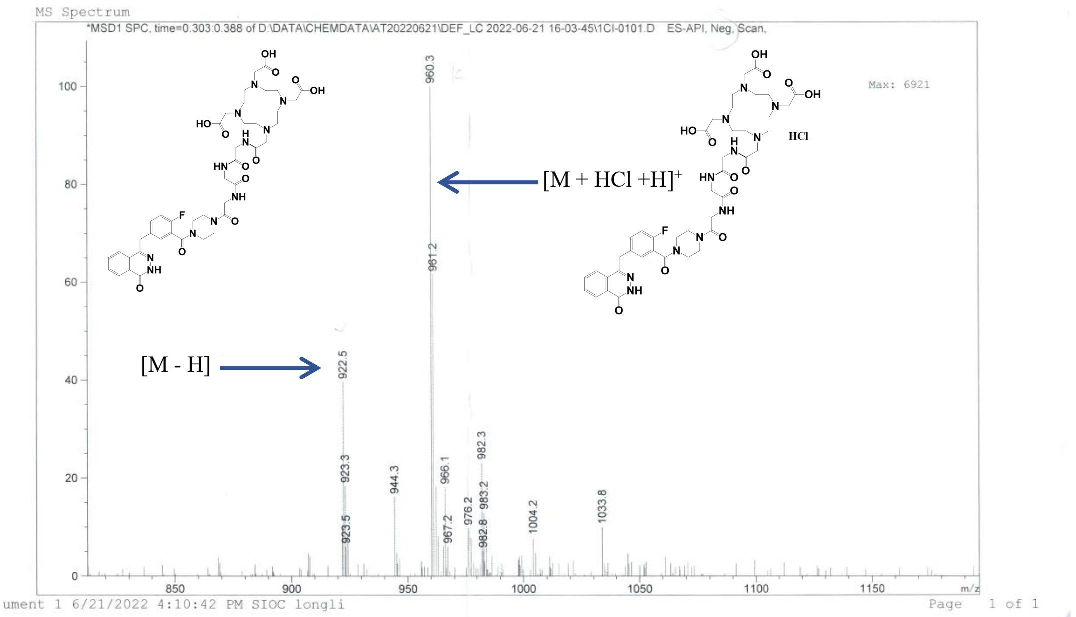

Fig. S29 MS spectrum of compound **DOTA-(Gly)$_3$-Olaparib**

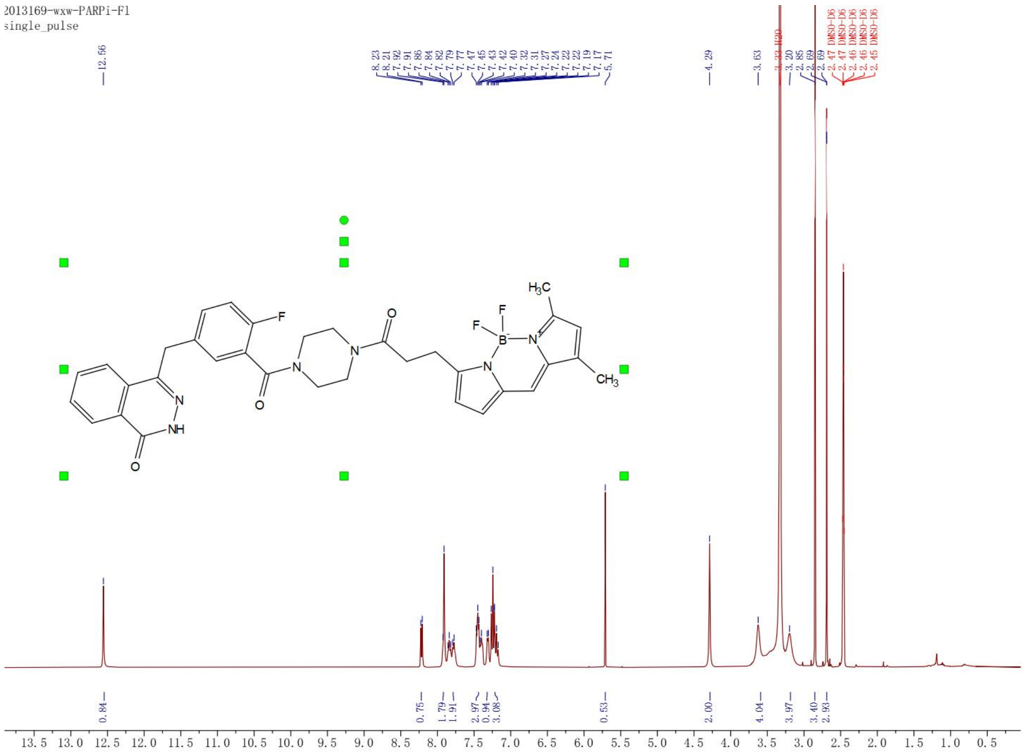

Fig. S30 ^{1}H NMR of compound **FL-Olaparib**
